# Dicyemida and Orthonectida: Two Stories of Body Plan Simplification

**DOI:** 10.3389/fgene.2019.00443

**Published:** 2019-05-24

**Authors:** Oleg A. Zverkov, Kirill V. Mikhailov, Sergey V. Isaev, Leonid Y. Rusin, Olga V. Popova, Maria D. Logacheva, Alexey A. Penin, Leonid L. Moroz, Yuri V. Panchin, Vassily A. Lyubetsky, Vladimir V. Aleoshin

**Affiliations:** ^1^Institute for Information Transmission Problems, Russian Academy of Sciences, Moscow, Russia; ^2^A.N. Belozersky Institute of Physico-Chemical Biology, Lomonosov Moscow State University, Moscow, Russia; ^3^Faculty of Bioengineering and Bioinformatics, Lomonosov Moscow State University, Moscow, Russia; ^4^Faculty of Biology, Lomonosov Moscow State University, Moscow, Russia; ^5^Skolkovo Institute of Science and Technology, Moscow, Russia; ^6^Department of Neuroscience, McKnight Brain Institute, University of Florida, Gainesville, FL, United States

**Keywords:** Mesozoa, Dicyemida, Orthonectida, genome, mitochondrial DNA, phylogeny

## Abstract

Two enigmatic groups of morphologically simple parasites of invertebrates, the Dicyemida (syn. Rhombozoa) and the Orthonectida, since the 19th century have been usually considered as two classes of the phylum Mesozoa. Early molecular evidence suggested their relationship within the Spiralia (=Lophotrochozoa), however, high rates of dicyemid and orthonectid sequence evolution led to contradicting phylogeny reconstructions. Genomic data for orthonectids revealed that they are highly simplified spiralians and possess a reduced set of genes involved in metazoan development and body patterning. Acquiring genomic data for dicyemids, however, remains a challenge due to complex genome rearrangements including chromatin diminution and generation of extrachromosomal circular DNAs, which are reported to occur during the development of somatic cells. We performed genomic sequencing of one species of *Dicyema*, and obtained transcriptomic data for two *Dicyema* spp. Homeodomain (homeobox) transcription factors, G-protein-coupled receptors, and many other protein families have undergone a massive reduction in dicyemids compared to other animals. There is also apparent reduction of the bilaterian gene complements encoding components of the neuromuscular systems. We constructed and analyzed a large dataset of predicted orthologous proteins from three species of *Dicyema* and a set of spiralian animals including the newly sequenced genome of the orthonectid *Intoshia linei*. Bayesian analyses recovered the orthonectid lineage within the Annelida. In contrast, dicyemids form a separate clade with weak affinity to the Rouphozoa (Platyhelminthes plus Gastrotricha) or (Entoprocta plus Cycliophora) suggesting that the historically proposed Mesozoa is a polyphyletic taxon. Thus, dramatic simplification of body plans in dicyemids and orthonectids, as well as their intricate life cycles that combine metagenesis and heterogony, evolved independently in these two lineages.

## Introduction

In spite of more than one hundred years of studies, the evolutionary relationships of the Mesozoa are still elusive. The name of this taxon reflects the traditional view of mesozoans as organisms with intermediate organization between unicellular protozoans and multicellular metazoans (Van Beneden, 1876; Hyman, 1940). Indeed, the two groups of microscopic parasitic invertebrates, the Dicyemida, and Orthonectida, display a remarkably simple morphological organization and a nearly complete absence of tissues and organs (Malakhov, 1990). Adult dicyemids inhabit the renal sacs of cephalopod mollusks and consist of just about 40 somatic cells, lack recognized muscular, nervous, sensory cells, and the organs typical for eumetazoans (Furuya et al., 2004). Dicyemids do not have a morphologically recognized basal membrane (Czaker, 2000), and never develop “true” tissues throughout their complex life cycle (Furuya and Tsuneki, 2003). The trophic stage of orthonectids is a syncytial plasmodium, which resides inside the invertebrate host and generates ephemeral ciliated organisms that exit the host for reproduction (Slyusarev, 2008). These organisms are composed of several hundred somatic cells without anatomically recognized digestive, circulatory, or excretory systems. Before the discovery of muscular and nervous systems in the swimming stages of orthonectids (Slyusarev and Starunov, 2015), they were thought to have a planula-like organization and were grouped with dicyemids in the Mesozoa as multicellular animals with an incredibly simple body plan, perhaps – the simplest among all Metazoa, and comparable to placozoans.

Intricate life cycles of dicyemids and orthonectids exhibit the alternation of asexual and sexual generations, termed metagenesis. Ameiotic generative cells (agametes) develop inside the dicyemid axial cell and later produce the next vermiform generation possessing gametic cells that undergo self-fertilization. In orthonectids, agametes develop inside the parasitic plasmodium and produce the free-living diecious (or hermaphroditic) generation (Cheng, 1986; Slyusarev, 2008). The phenomenon of successive sexual parthenogenetic and amphimictic generations is termed heterogony. In this sense, orthonectids and dicyemids as well as parasitic flatworms combine metagenesis and heterogony in their life cycles. Particularly, trematode sporocysts and rediae that parasitize gastropod mollusks produce the next generation from ameiotic generative cells (Dobrovolskij and Ataev, 2003; Ataev, 2017). Similarities in life cycles for long sustained the hypothesis about close relationships of dicyemids and orthonectids with digenetic trematodes. On the other hand, intracellular localization of generative cells relates dicyemids and orthonectids with myxozoans rather than trematodes. Such intricate combination of traits makes life strategies in dicyemids and orthonectids unique among animals.

The phylogenetic affinity of dicyemids and orthonectids has been called into question on the grounds of morphology (Kozloff, 1990; Brusca and Brusca, 2003; Ruppert et al., 2004). Molecular data conclusively demonstrated that both dicyemids and orthonectids are in fact bilaterians (Katayama et al., 1995; Hanelt et al., 1996; Pawlowski et al., 1996; Aruga et al., 2007) and belong to the diverse clade of Lophotrochozoa (=Spiralia) (Kobayashi et al., 1999, 2009; Petrov et al., 2010; Suzuki et al., 2010; Mikhailov et al., 2016; Lu et al., 2017; Schiffer et al., 2018), thus implying that their simple organization evolved as the result of their parasitic lifestyle.

In molecular phylogenetic analyses, dicyemid and orthonectid lineages display extremely high levels of divergence, and their exact placement among the spiralians remains ambiguous and potentially prone to long branch attraction artifacts. Complicating the matter is the uncertainty in relationships between other spiralian taxa, including the Annelida, Mollusca, Nemertea, Brachiopoda, Entoprocta, and Bryozoa (Kocot, 2016). Recent phylogenomic analyses lead to conflicting conclusions regarding the mesozoan phylogeny. Lu et al. (2017) using a dataset of 348 orthologs (58,124 alignment positions) from 23 spiralian species, including an orthonectid and a dicyemid, report the monophyly of the Mesozoa either as a sister group to the Rouphozoa (Platyhelminthes + Gastrotricha) or within the Gastrotricha. Alternatively, Schiffer et al. (2018) using a dataset of 469 orthologs (190,027 alignment positions) from 29 spiralian species, including an orthonectid and two dicyemids, conclude that Orthonectida and Dicyemida evolved independently within the Lophotrochozoa, with the orthonectids exhibiting clear affinity to annelids, and dicyemids occupying an isolated position within Lophotrochozoa. Here, we obtained transcriptomic and genomic data for dicyemid species to resolve this contradiction.

The dicyemid genome is distinguished by uncommon features, such as the genome rearrangements during the life cycle and generation of circular DNAs (Noto et al., 2003), including those that encode mitochondrial proteins and rRNAs (Watanabe et al., 1999; Catalano et al., 2015). It is not yet established if the mitochondrial protein-coding genes are encoded only by small circular DNA molecules (Watanabe et al., 1999) or whether they are produced during the dicyemid development from a precursor mitochondrial DNA with a more typical metazoan organization (Awata et al., 2006). Using high-throughput genomic sequencing we sought to find any properties of dicyemid sequences that would reveal their genome organization. We also estimated the extent of gene losses due to the simplification of dicyemid morphological organization, and analyzed whether losses in particular gene families and regulatory pathways are the same or different compared to an orthonectid *Intoshia linei*.

**Table 1:** Assembly statistics.

	*Dicyema* sp. genomic	*Dicyema* sp. 454	*Dicyema* sp.	**Dicyema* japonicum*	*Dicyema* sp.genomic filtered^∗^	*Dicyema* sp. 454 filtered^∗∗^	*Dicyema* sp. filtered^∗∗^	*Dicyema japonicum* filtered^∗∗^
Assembly size (bp)	858,248,066	19,669,371	64,598,656	44,413,963	N/A	N/A	N/A	N/A
Contigs/transcripts (>500 bp)	939,453	22,115	52,176	29,091	N/A	N/A	N/A	N/A
Predicted genes/peptides	984,055	12,379	22,286	11,330	21,842	11,726	21,656	11,233
Complete BUSCOs, eukaryota_odb9	77.6%	65.0%	82.2%	85.1%	71.3%	62.0%	80.2%	84.2%
Complete and single-copy BUSCOs (S)	74.6%	63.0%	77.9%	82.5%	68.3%	60.7%	76.6%	81.2%
Complete and duplicated BUSCOs (D)	3.0%	2.0%	4.3%	2.6%	3.0%	1.3%	3.6%	3.0%
Fragmented BUSCOs (F)	14.2%	22.8%	9.2%	6.6%	11.6%	23.1%	10.2%	6.6%
Missing BUSCOs (M)	8.2%	12.2%	8.6%	8.3%	17.1%	14.9%	9.6%	9.2%

*^∗^Genomic predictions were filtered by retaining only hits to the InterPro database and cleaned from the cephalopod contamination with BLAST searches against the NCBI nr database. ^∗∗^Transcriptome assemblies were filtered with BLAST searches against the RefSeq database as detailed in Materials and Methods, Section “Assembly and Filtering of Dicyemid Sequences.”*

## Results and Discussion

### Genomic Sequencing and Assembly of *Dicyema* sp.

Direct assembly of a dicyemid genome from whole DNA extracts using standard approaches is an extremelly challenging problem due to drastic genome rearrangements that occur in dicyemids during development. Previous studies have demonstrated that somatic cells of dicyemids undergo drastic genome rearrangements and chromatin elimination (Noto et al., 2003), and suggested that selective and whole genome amplification takes place at different stages of their development (Awata et al., 2006). Accordingly, the sequencing of whole DNA extracts from *Dicyema* sp. resulted in a highly fragmented assembly with uneven coverage and N50 of 942 bp, where the largest contig was only around 20 Kb. The total size of the assembly is 858 Mbp in nearly 1 million contigs over the length of 500 bp, and includes contaminating cephalopod sequences. Due to significant genetic difference between the dicyemid host *Enteroctopus dofleini* and the available genomic sequence of *Octopus bimaculoides*, the filtering of the assembly was performed at the level of predicted gene products. Only predictions identifiable by hits against the InterPro database were retained for the subsequent comparative analyses and filtered from the cephalopod contamination using the best hit approach with BLAST searches against the NCBI nr database. Out of 38,410 predictions with InterPro hits, 43% were discarded as contamination, resulting in 21,842 putative dicyemid genes with 71% complete and 12% fragmented universal eukaryotic orthologs evaluated by BUSCO (Table 1). Similar values are obtained for gene predictions after normalizing on the number of BUSCOs found in at least one filtered transcriptome: 76% complete and 12% fragmented. The total percentage of BUSCOs recovered by at least one sequencing library, including genomic and transcriptomic filtered data, approaches values seen in typical metazoan genomes: 91% complete and 3% fragmented. For all analyses in Sections 2.4–2.11 we used original genomic data on *Dicyema* sp., and the three transcriptomes, including the two originally obtained and the one of *Dicyema japonicum* available from the published source (Lu et al., 2017).

**FIGURE 1:**
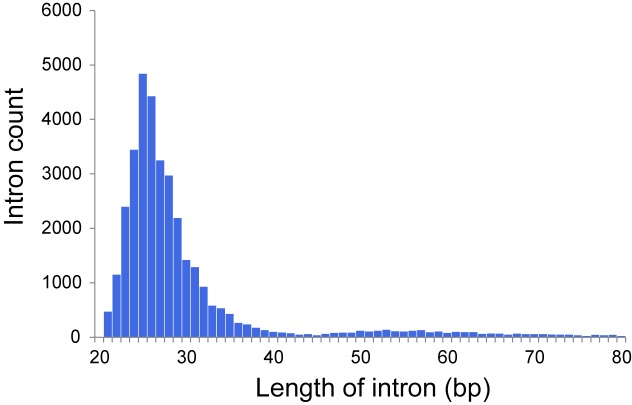
Distribution of intron lengths in predicted genes of *Dicyema* sp.

The dicyemid genes display miniaturization of spliceosomal introns – the median length of introns is 27 bp, and approximately two thirds of predicted introns are under the length of 30 bp (Figure 1). This agrees with an earlier survey that revealed extreme intron shortening in a set of 40 genes from *D. japonicum* (Ogino et al., 2010). The estimated intron density in *Dicyema* sp. is 4.9 introns/gene for predictions with intact start and stop codons, which is also similar to the 5.3 introns/gene reported for *D. japonicum*. Similar value of intron density is seen in the genome of orthonectid *I. linei* (Mikhailov et al., 2016). Notably, the orthonectid genes also harbor short spliceosomal introns, but the majority of its introns are longer than 30 bp, and the median size is 57 bp, considerably exceeding the intron lengths observed in dicyemid genes.

### “Circular” Contigs in Genomic Assembly of *Dicyema* sp.

**FIGURE 2:**
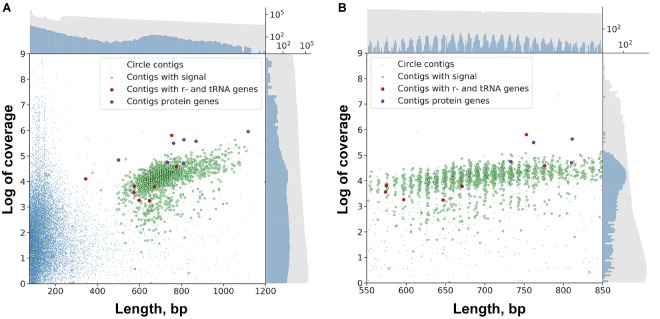
Scatter plot of circular contigs in the *Dicyema* sp. genome. Different markers stand for circles with the specific signal and with mitochondrial genes (see Text for clarification). **(A)** The distributions for both length and coverage logarithm are non-uniform — there is a pool of “long” contigs with a high level of coverage in the set. **(B)** The distribution of “long” contigs with a high level of coverage is, in turn, non-uniform; the distance between two adjacent peaks is approximately 10.44 bp. On both scatterplots the upper axis shows the distribution of contig lengths, and the right axis shows the coverage. Blue dots correspond to circular contigs, and green dots are linear contigs. The scale is logarithmic. Both the coverage and length of circular contigs is obviously less uniform compared to those of linear contigs.

Using the genomic assembly we have identified 24,065 “circular” contigs (see section “Materials and Methods”). The distribution of circular contig lengths in the assembly is multimodal (Figure 2A). The first abundant pool of sequences is formed from contigs less than 500 bp. The second pool, which includes sequences of a length over 500 bp, consists of 3,220 contigs with the median length of 702 bp. The properties of the sequences in this pool (such as length and abundance) are consistent with previous data of DNA gel electrophoresis, EM and PCR experiments (Noto et al., 2003), which supports the conjecture that these sequences are circular DNA rather than direct repeats. “Short” circles (up to 500 bp length) were shown to possess 38.1% low complexity regions, while “long” circles – only 2.9%. This observation might suggest that a fraction of predicted short circles represents direct repeats. Following this rationale, we considered the two sub-pools separately in analyses.

**FIGURE 3:**
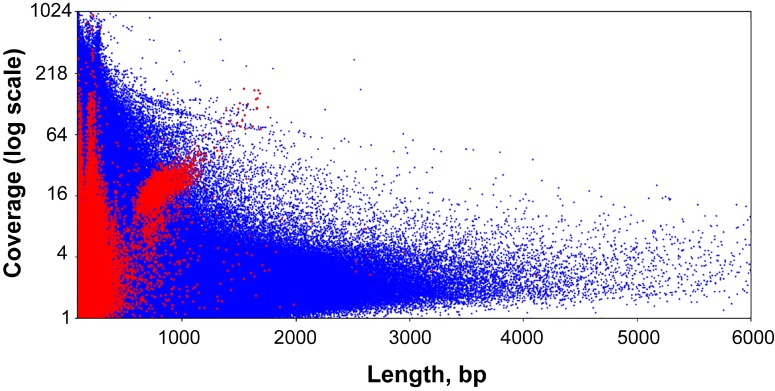
Scatter plot of the entire set of *Dicyema* sp. genome assembly contigs. Blue dots are linear contigs, and red dots are circular ones.

The lengths of sequences from the second pool of circular contigs are distributed non-uniformly which is particularly evident within the 600–800 bp range (Figure 2B). The average distance between two adjacent peaks of this distribution is 10.44, which closely corresponds to the number of base pairs in one turn of B-DNA. Multimodal distribution was also observed (Kolmogorov–Smirnov test *p*-value is 0.999) when performing assembly with the varying *k*-mer size (55 or 77) and with another assembly method (Supplementary Figure S1). The presence of this pattern is unexpected, and presumably could be attributed to the greater stability of circles or tendency to circularize for molecules with an integer amount of turns of a relaxed form of DNA. A similar effect has also been observed in short (<200 bp) sequences as a result of rolling circle replication bias (Joffroy et al., 2018). This distribution can result from the random ligation of linear molecules cut from the genome as it leads to the reduction in DNA supercoiling. Alternatively, replicating mini-circular DNA molecules can be selected in length to reduce their supercoiling. Figure 3 shows that the coverage value for “long” circular contigs is not lower than for linear ones, which casts doubt on the proposed diminution of circular molecules during ontogenesis.

**FIGURE 4:**
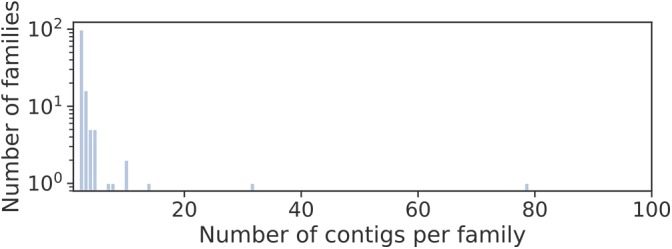
Distribution of families of long circular contigs in *Dicyema* sp. vs. the number of members per family. To detect families, *blastn* searches of all long (>500 bp) circular contigs against themselves were conducted. The graph elements are contigs, and the edge weight is hits *E*-value. All edges with the weight greater than 1e-10 were deleted, and contigs in one connected component are considered a family.

Long circular contigs are predominantly not similar in nucleotide sequences. Only 15% of them have at least one fairly similar contig, and only three families of contigs unite more than 10 members (Figure 4).

**FIGURE 5:**
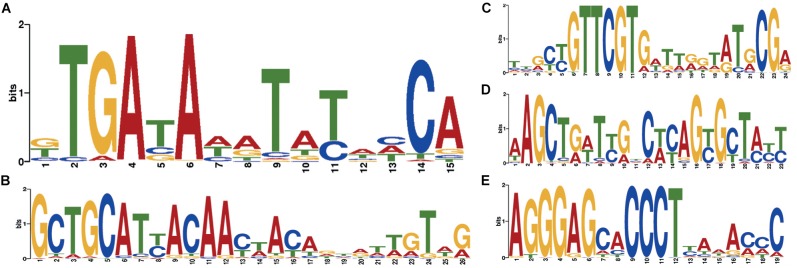
Motifs specific to *Dicyema* sp. circular contigs. **(A)** Signal found in the majority of contigs. **(B–E)** Highly conserved signals found in a group of circular contigs of high length and coverage; **(B)** found in 541 contigs; **(C)** found in 284 contigs; **(D)** found in 234 contigs; **(E)** found in 438 contigs. All counts are provided at a *p*-value < 10^−4^.

Two independent motif detection methods (Bailey and Elkan, 1994; Rubanov et al., 2016) have been applied to the circles of length 600–800 bp with a coverage logarithm of over 3 (2,031 sequences). In 1,871 sequences (92.12% of sequences in the analysis) common motifs have been found (*E*-value: 4.8e-82, see Figure 5A).

At a *p*-value < 10^−5^, the most common motif occurs on average once every 874 bp in “long” circles and every 21,863 bp throughout the entire assembly (statistical significance of the difference provided by the chi-squared criterion: *p*-value < 0.001). The search for highly conserved sequences in various subsets of genome sequences has demonstrated that less common motifs with high information content can also be found in circles (Figure 5B–E).

The search for conserved domains in circular contigs recovered only domains of mtDNA-encoded proteins (10 conserved domains, 13 contigs including paralogs). These sequences are presumably transcribed as they are also found in the RNA-seq data (*blastn* search, *E*-value < 1e-30).

### Mitochondrial DNA of *Dicyema* sp.

**FIGURE 6:**
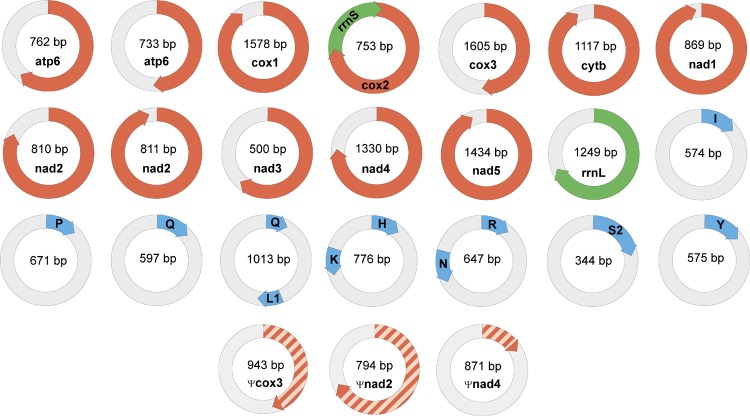
Mini-circles coding mitochondrial genes in *Dicyema* sp. Lenght of each minicircle is marked in its center. Each minicircle has a coding region with gene name and transcription orientation, and a non-coding region (in light gray). Protein-coding genes marked in red, rRNA genes marked in green, and tRNA genes marked in blue. tRNA genes indicated by a one-letter amino acid code.

Genomic data confirm the localization of mitochondrial genes of dicyemids on circular DNA molecules (Watanabe et al., 1999; Catalano et al., 2015). The search for mtDNA genes in the genomic data found 21 circles with length varying from 344 to 1605 bp. The following gene sequences were found: *cox1-3*, *cob*, *nad1-5*, *atp6*, *rrnL*, *rrnS*, *trnH*, *trnI*, *trnK*, *trnL1*, *trnN*, *trnP*, *trnQ*, *trnR*, *trnS2*, and *trnY* (Figure 6). In earlier studies the dicyemid mitochondrial contigs were found to carry either one protein coding gene (Watanabe et al., 1999) or a protein coding gene and a tRNA gene (Robertson et al., 2018). We found one circle that contains two genes – *cox2* and *rrnS*, and three circles that contain two tRNA genes each. Protein identity between mitochondrial predictions for *Dicyema* sp. and the earlier published *D. japonicum* (Robertson et al., 2018) varies from 39% (nad2) to 75% (cox1). The majority of mitochondrial genes can also be found in the transcriptomic data, except for *atp6* and *nad5*. The mtDNA circles also contain the motif described above (Figure 5A) (*p*-value < 10^−5^).

The *nad2* and *atp6* genes were found in two different variants in the genomic data. Two paralogs of nad2 with lengths of 215 and 252 amino acids have 42% identity at the amino acid level. Two paralogs of atp6 with lengths of 117 and 149 amino acids have 89% identity at the amino acid level, and share two long deletions with other dicyemids. These deletions are specific for dicyemids and are not found in other taxa including Orthonectida. Both of dicyemid deletions are located outside of the transmembrane helices – the first one with the length of 16 amino acids is located in the region facing the mitochondrial matrix and the second one with the length of 17 amino acids is located in the region facing the intermembrane space, according to the alignment of atp6.

We predicted 11 mitochondrial tRNA genes in *Dicyema* sp. including two paralogs of glutamine tRNA gene (Supplementary Figure S2). Both dicyemid glutamine tRNAs have similar secondary structures and lack a T-arm. Dicyemid arginine tRNA also lacks a T-arm and lysine tRNA lacks a D-arm. Other mitochondrial tRNAs maintain the typical clover leaf structure, although several tRNA genes have single nucleotide insertions and/or non-complementary pairs in stems. Experimental evidence is needed to confirm all the predicted tRNA genes, as well as decisions whether numerous not listed tRNA-like sequences with *p*-value below the threshold are functional genes.

Read mapping to the genomic assembly revealed no read pairs that would facilitate mtDNA scaffolding. Whenever one read from a pair would map to the circular mitochondrial contig, the other would map to the same contig or have a sequence of low complexity. Thus, our genomic data fails to confirm the hypothesized existence of an unprocessed mtDNA precursor, which would generate the mtDNA circles (Awata et al., 2006).

The presence of common sequence motifs in circles with mtDNA genes and without them seems to be surprising. It can be interpreted as a consequence of a similar mechanism of generation and maintaining of circles irrespective of their function.

The partitioning of mtDNA into circular molecules is a rare feature for the animal mitochondrial genomes (Odintsova and Yurina, 2005; Burger et al., 2012; Kolesnikov and Gerasimov, 2012; Smith and Keeling, 2015; Lavrov and Pett, 2016, for review). In bilaterians, the mtDNA is fragmented into a large number of mini-chromosomes in the cyst-forming nematodes *Globodera* spp. (Armstrong et al., 2000; Gibson et al., 2007) and sucking lice (Shao et al., 2009). Notably, the mitochondrial DNAs from orthonectids *Intoshia linei*, *Intoshia variabili*, and *Rhopalura ophiocomae* retain typical structure for metazoans and encode the full set of mitochondrial genes on a single circular molecule (Robertson et al., 2018; Bondarenko et al., 2019). The reason why the mitochondrial *Dicyema* spp. genome is fragmented is unknown. Earlier, the fragmentation of the mitochondrial genome of sucking lice was considered (Shao et al., 2009) as an adaptation to the high rate of molecular evolution, which is even more characteristic of *Dicyema* spp. It is possible that under conditions of high mutagenesis, a set of uncorrupted genes is easier to assemble from individual than concatenated molecules.

**FIGURE 7:**
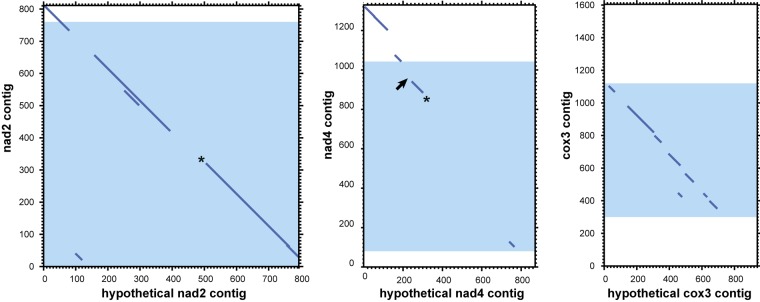
Dotplots of local alignments of circular contigs against genes *nad2*, *nad4*, and *cox3*. Blue zones represent mitochondrial genes with the open frame. Asterisks mark homologs with stop codons, arrows mark frameshifts. Results obtained with LAST (Kiełbasa et al., 2011) (threshold *E*-value for local alignment 0.008).

Analysis of mitochondrial DNA suggests an explanation of the multiple observed circular contigs. For searches with the *tblastx* algorithm we used proteins from the annotated mitochondrial contigs as the query and all 3,220 “long” circle contigs as the database. The searches returned many circular contigs that encode highly diverged genes *cox3*, *nad2*, and *nad4* (Figure 7). The *cox3* homolog is largely diverged, while *nad2* and *nad4* contain stop codons and frame shifts. These contigs therefore represent mitochondrial pseudogenes.

Previous publications and our new data confirm the presence of two unusual features of the *Dicyema* genome. First, mitochondrial genes in *Dicyema* are not located on a single long DNA molecule as in most animals, but are partitioned into smaller circular molecules. The second interesting feature of *Dicyema* is the presence of thousands of non-coding circular DNA sequences. Both types of circular DNA molecules fall in a similar range of size and coverage in DNA assembly and bear a common set of similar 12–20 bp DNA patterns, which might be hypothetical signal sequences. We assume that all circular DNAs in *Dicyema* may have a common origin, although experimental evidence is necessary. We speculate that the presence of multiple mtDNA mini-rings instead of one long molecule might have produced serious problems in mitochondrial division. This requires special mechanisms to correct distribution of multiple minicircular DNA molecules upon mitochondrion division so that both descendants would obtain a complete set of genes. Specific signal patterns like the ones we observe could be used to support circular mtDNA duplication, their protection against elimination or their correct distribution between descendent mitochondria. When such mechanisms are established, it is possible that rings carrying mutated (pseudo)genes or other selfish non-coding DNA circular elements acquire similar signal sequences that ensure their preservation in a similar way as with parasitic mobile genetic elements.

### Homeobox Transcription Factors

Homeodomain (homeobox) transcription factors are crucial regulators of animal development that play central roles in tissue differentiation and axial body patterning. Bilaterian genomes encode from over 300 to around 60 homeobox genes. The genome of orthonectid *I. linei* was found to possess one of the smallest repertoires of homeoboxes (Mikhailov et al., 2016), which matches the reduced complexity of their organization. To determine how the extreme simplification of body plan seen in dicyemids relates to their homeobox gene content we searched for these genes in the dicyemid genomic and transcriptomic data. For analyses with HMMER, we used gene predictions coming from the genomic assembly of *Dicyema* sp. (PRJNA527259; designated as *Dicyema* sp. 1) and transcriptome assemblies of *Dicyema* sp. (SRR827581; designated as *Dicyema* sp. 2) and *Dicyema japonicum* (DRR057371). HMMER searches using the homeodomain profile identified 38, 39, and 55 homeoboxes in the three dicyemids after filtering out contaminating cephalopod sequences. Phylogenetic inference suggests that dicyemid homeoboxes form up to 39 families, and each dicyemid was found to contain 31 or 34 families (Table 2). A high level of sequence divergence complicates classification of the dicyemid homeoboxes. Although most dicyemid sequences could be assigned to one of the homeobox classes (Zhong and Holland, 2011), their attribution to known families is inconclusive.

**Table 2:** The list of homeodomain transcription factors in three species of Dicyemida.

	*Dicyema* sp. 1	*Dicyema* sp. 2	*Dicyema japonicum*
**Class ANTP**	9	13	10
Subclass HOXL	5	8	7
Family Hox6-8 or ‘central’ Hox genes	3	3	3
Dicyemid ‘central’ Hox group 1 (DoxC)	1	1	1
Dicyemid ‘central’ Hox group 2 (DoxC paralog)	1	1	1
Dicyemid ‘central’ Hox group 3	1	1	1
Family Hox9-13(15) or ‘posterior’ Hox genes	0	0	1
Dicyemid HOXL group	1	4	2
Family Evx (even-skipped)	1	1	1
Subclass NKL	4	5	2
Family Dlx (distal-less)	1	1	0
Nk2 genes (families Nk2.1 and Nk2.2)	3	4	2
Dicyemid Nk2 group 1	1	2	1
Dicyemid Nk2 group 2	2	2	1
Other ANTP	0	0	1
**Class PRD**	5	6	6
Family Pax 4/6	1	1	1
Family Otx (orthodenticle)	1	1	1
Dicyemid PRD group 1	1	3	1
Dicyemid PRD group 2	1	0	1
Dicyemid PRD group 3	0	1	1
Dicyemid PRD group 4	1	0	1
**Class POU**	5	7	1
Dicyemid POU group 1	1	1	0
Dicyemid POU group 2	1	1	1
Dicyemid POU group 3	1	1	0
dicyemid POU group 4	1	1	0
Dicyemid POU group 5	1	3	0
**Class LIM**	4	9	4
Family Lhx6/8	1	2	0
Family Lhx2/9	0	0	1
Family Isl	1	1	2
Dicyemid LIM group 1	0	1	1
Dicyemid LIM group 2	1	4	0
Dicyemid LIM group 3	1	1	0
**Class SINE**	3	5	5
Family Six3/6	1	1	1
Dicyemid SINE group 1	1	1	2
Dicyemid SINE group 2	1	1	1
Dicyemid SINE group 3	0	2	1
**Class CUT**	2	2	2
Family Onecut	2	2	2
Dicyemid Onecut group 1	1	1	1
Dicyemid Onecut group 2	1	1	1
**Class TALE**	8	7	9
Family Pbx	1	1	1
Family Tgif	1	0	0
Dicyemid TALE group 1	3	1	2
Dicyemid TALE group 2	1	1	2
Dicyemid TALE group 3	1	2	1
Dicyemid TALE group 4	1	2	3
**Class ZF**	2	2	1
Family Zfhx	2	2	1

Phylogenetic inference with PRD class homeoboxes reveals six dicyemid families, including the previously identified orthologs of the Pax6 and Otx (Aruga et al., 2007; Kobayashi et al., 2009). Five dicyemid sequence groups were found among the LIM homeoboxes, two of which are grouped with the Lhx6/8 and Islet family sequences. An additional LIM class homeobox of the Lhx2/9 family was found in *D. japonicum*, but could neither be confirmed by data from the other dicyemids nor discarded as contamination. Another five dicyemid gene groups belong to the POU class homeoboxes, but branch outside of any known families. The dicyemids form at least 5 TALE class sequence groups, with one group branching within the Pbx family. An additional single member of the TALE Tgif family was found only in the genomic data. Four SINE class families were found among the dicyemid sequences, with one grouping with the Six3/6 family. The dicyemids also possess a zinc finger homeobox, and a group of Onecut family sequences, which can be subdivided into 2 dicyemid-specific families.

**FIGURE 8:**
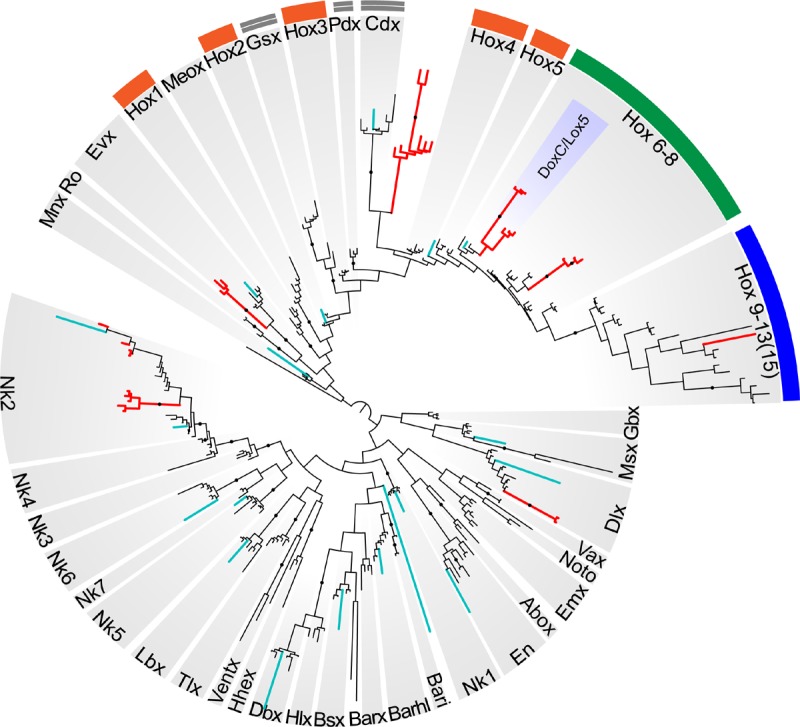
Bayesian tree of the ANTP class homeodomain sequences from *Homo sapiens*, *Drosophila melanogaster*, *Capitella teleta*, *Octopus bimaculoides*, *Intoshia linei*, and three dicyemids: *Dicyema* sp. 1, *Dicyema* sp. 2, and *Dicyema japonicum*. The dicyemid sequences are given in red, and the orthonectid homeoboxes are labeled with teal color. The groups of anterior Hox genes (Hox1-5) are outlined in orange, the central Hox genes (Hox6-8) – in green, and the posterior Hox genes (Hox9-13) – in blue; ParaHox orthologs (Gsx, Pdx, and Cdx) are marked with a double line. The dicyemid DoxC/Lox5 genes are labeled inside the group of central Hox genes. Nodes with ≥0.95 posterior probability are marked with black dots.

Reconstructions with the ANTP class homeoboxes recover 8 dicyemid sequence groups (Figure 8). Three of these groups fall within the central Hox sequences. One of the dicyemid central Hox groups corresponds to orthologs of DoxC – a dicyemid member of the spiralian Lox5 family (Kobayashi et al., 1999), which was shown to have an expression pattern consistent with defining anterior–posterior boundaries in the developing dicyemids (Aruga et al., 2007). The analysis suggests that dicyemids possess another member of the Lox5 family – all three dicyemids were found to encode a paralog of the DoxC. The paralog displays greater sequence divergence, but similar to other members of the family retains a Lox5-specific motif flanking the C-terminus of the homeodomain (de Rosa et al., 1999). The third dicyemid group within the central Hox sequences is found outside the Lox5 family and tends to group with the Lox2/Lox4 families, but beyond that does not lend itself to classification. A single posterior type Hox gene was found in *D. japonicum*, but once again could not be verified using other dicyemid sequences or rejected as contamination by BLAST searches. The dicyemid ANTP class homeoboxes also include members of the Evx, Dlx, and Nk2 families, and a conspicuous group of Hox-like sequences (Figure 8). Sequences within the dicyemid Hox-like assemblage share a common ancestor and retain a YPWM motif, which is essential for binding Hox cofactors (Prince et al., 2008), but this group is too divergent to be classified with any family, and is placed with the longest branch of Hox-like genes – the ParaHox Cdx family.

The survey of dicyemid genes suggests that overall they possess fewer homeoboxes than the orthonectid *I. linei* and their sequences are also markedly more diverged. Unlike the orthonectid, no ParaHox or anterior Hox families could be readily identified in the dicyemid data. Reduction of homeobox transcription factors in dicyemids is consistent with extreme simplification of their body plan. Unexpectedly, the dicyemids also experience several lineage-specific expansions of homeoboxes, notably the duplication of central Hox gene DoxC, which opposes the general trend of regulatory gene loss.

### Basement Membrane

The basement membrane is a structure that enables the compartmentalization of cells to form tissues and organs. It is present in the majority of metazoans, with exception of sponges, placozoans, and acoelomorphs. The reported loss of a morphologically recognized basement membrane in dicyemids would indicate unprecedented simplification in this animal group. Even though this topic has been studied (Czaker, 2000), it is still unclear whether dicyemids have a basement membrane during any of their life cycle stages. The basement membrane consists of a set of “basement membrane toolkit” proteins, but the most important are collagen IV and laminin (Fidler et al., 2017). Both laminin and type IV collagen are multi-domain proteins that include specific domains (LamNT for laminin and C4 in the case of collagen type IV) and non-specific domains (EGF-like and other). The BLAST and Pfam searches showed that these domains of canonical molecules forming basement membrane are absent from the sequenced genome of *Dicyema* sp., therefore supporting the proposed secondary loss of this trait in dicyemids. The apparent absence of the recognized basement membrane is parallel with a reported loss of muscular and nervous systems in these animals. Indeed, in bilaterians, the basement membrane supports the maintainance of the muscular and nervous system architecture, their development and compartmentalization, and supporting growth factor signaling gradients among other functions.

The complete life cycle of dicyemids is not entirely understood, and more complex structures of transitional obscure life forms of these organisms are not excluded. An unknown stage can potentially exist between the infusorioform larvae that exits the host and the vermiform embryos found in cephalopods. The lack or reduced representation of genes encoding key elements of the basement membrane or other mediators of organ formation further supports the idea that dicyemids are secondarily simplified to an outstanding state.

### Membrane Receptor Proteins

Cell surface membrane receptors act in cell signaling and allow communication between the cell and the extracellular space. Their diversity reflects the complexity of the organism and its ability to respond to different external signals. The number of genes encoding receptor proteins in dicyemids is exceptionally low. We found only two PF00001 domain hits corresponding to the 7 pass transmembrane receptor proteins of rhodopsin family in *Dicyema* sp. This family of G-protein-coupled receptors (GPCRs) is ubiquitously present and abundant in metazoans and contains tens to hundreds of members in different species. The minimum number of the rhodopsin family genes (six per genome) is detected in the sponge *Amphimedon queenslandica*; even the genome of the simplified orthonectid *I. linei* contains 32 genes of the rhodopsin family. The actual specificity of these GPCRs proteins is unknown, although their BLAST search shows best similarity to the rhodopsin family neuropeptide receptors from other animals. Four proteins from another GPCR 7 pass transmembrane receptor family – secretin family (PF00002) were predicted in the *Dicyema* sp. data. This is fewer than in most metazoans yet some flatworms have even fewer (Zamanian et al., 2011), and the Orthonectida have no such proteins. We found one putative metabotropic glutamate receptor with a PF00003 domain. Curiously, this metabotropic glutamate receptor also contains a (LIVBP)-like domain that is characteristic of ionotropic glutamate receptors. Two ionotropic glutamate receptors (iGluRs) that are ligand-gated ion channels activated by the neurotransmitter glutamate with Lig_chan (PF00060) domain were identified in *Dicyema* sp. One of them with a PF10613 (Lig_chan-Glu_bd) and another with PF01094 (ANF_receptor). Thus, both distinct types of glutamate receptors (ionotropic and metabotropic types) are present in *Dicyema* sp. It is well known that glutamate is often associated with non-neuronal signaling and is highly abundant in some animals that lack nervous systems (such as sponges and *Trichoplax*). Previously, we reported that iGluRs are absent in the genome of orthonectid (Mikhailov et al., 2016). Glutamate receptors are also found in plants and many other eukaryotes outside Metazoa (Turano et al., 2001).

Another big group of ionotropic receptors is the Cys-loop ligand-gated ion channel superfamily that is composed of nicotinic acetylcholine, GABA-A, GABA-A-ρ, glycine, 5-HT3, and zinc-activated (ZAC) receptors. We found 8 genes for this superfamily in *Dicyema* sp., identified by the specific transmembrane region domain (PF02932) and the ligand binding domain (PF02931). All these receptors are predicted to be nicotinic acetylcholine-like receptors.

### Ion Channels

**FIGURE 9:**
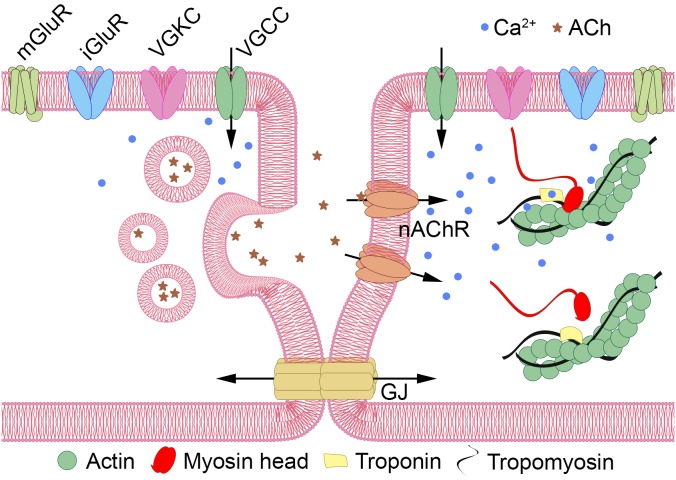
Hypothetical schema of intercellular communication in Dicyemida. Dicyemids have no recognizable neurons and muscles, and yet they have key elements of the neuromuscular system. Metabotropic (mGluRs) or ionotropic glutamate receptors (iGluRs) activate the “presynaptic” cell **(left)**. Voltage-gated tetrameric calcium (VGCCs) and potassium (VGKCs) ion channels generate propagating action potentials. Ca^++^ (blue dots) enters the cytoplasm via VGCCs and triggers the vesicular acetylcholine (ACh, shown by asterisks) release. Activation of nicotinic acetylcholine-like receptors (nAChR) increase Ca^++^ level in the “postsynaptic muscular” cell **(right)** directly or by depolarization of the plasma membrane and VGCC opening. Ca^++^ promote contractile elements activation via the Ca^++^ dependent troponin-tropomyosin-actin-myosin mechanism. Additional interaction of the two cells can occur via gap junctions (GJ).

Despite the reported absence of muscles and neurons, tetrameric ion channels that are often associated with cellular electrical excitability are present in *Dicyema* sp. in numbers similar to the orthonectid *I. linei* (33 and 36 sequences with PF00520, and 11 and 9 with PF07885 in *Dicyema* and the orthonectid, respectively). Although unlike Orthonectida no signatures for voltage-gated sodium ion channel (Na_trans_assoc PF06512) were detected in *Dicyema*, Pfam analysis (for Ca_chan_IQ PF08763) and reciprocal BLAST searches indicates the presence of voltage-gated calcium ion channels in this animal group. The presence of such channels together with tetrameric potassium ion channels implies that electrical excitability in the form of action potentials might exist in dicyemid cells. Figure 9 provides a hypothetical schema of the intercellular communication and an analog of the neuromuscular junction in dicyemids. This structure may potentially assemble from key predicted proteins typical to many other metazoans.

### Genes Encoding Putative Contractile/Muscular Elements

“True” muscle cells are absent in dicyemids and detection of the muscle-specific genes in these animals is interesting. Most of the core muscle proteins, including a type II myosin heavy chain (MyHC) motor protein was already present in unicellular eukaryotes before the origin of multicellular animals (Steinmetz et al., 2012). At the same time, the troponin complex appears to be a universal innovation of bilaterians. Troponin is a complex of three proteins (troponin C, troponin I, and troponin T). These proteins are detected in the dicyemid data by BLAST search, and the troponin domain PF00992 is found by Pfam search. The troponin complex is characteristic of skeletal and cardiac muscles, but not for smooth muscles. It appears that throughout the radical simplification in dicyemids that resulted in massive gene loss (including most of genes encoding the extracellular matrix ECM molecules) and in the absence of specialized muscle cells the troponins remain essential. The presence of troponins relates dicyemids to all other bilaterians with one remarkable exception – the orthonectid. In contrast to dicyemids and other bilaterians, the genome of orthonectid *I. linei* has no troponins despite having specialized muscles. Morphological data suggest that muscles in *I. linei* are similar to smooth muscles, so troponin was likely lost in *I. linei*, and its absence is a derived feature. At the same time another bilaterian hallmark – the myogenic regulatory factor (Myogenic Basic domain PF01586) – is present in the genome of *I. linei*, but was not detected in dicyemids. These findings support the mosaic evolution of many bilaterian traits, supporting the possibility of independent simplifications in these two parasitic lineages.

### Gap Junctions and Adhesion Molecules

Gap junctions are a distinct type of intercellular communication channels. In Metazoa, the gap junction proteins belong to two unrelated families – connexins and pannexins (also known as innexins). The connexins are only found in chordates, while the pannexin family is widespread in invertebrates. The presence of gap junctions and innexin/pannexins in dicyemids was demonstrated earlier by transmission electron microscopy (TEM) (Furuya et al., 1997) and molecular cloning (Suzuki et al., 2010). BLAST and Pfam searches with our dicyemid data detected 21 hits with the innexin/pannexin-specific Pfam domain (PF00876) and no connexins. The number of dicyemid pannexins is similar to other invertebrates (25 in *Caenorhabditis elegans*, 13 in *Drosophila melanogaster*). It appears that unlike the highly reduced chemical signaling, direct intercellular communication via gap junctions is conserved in dicyemids.

Other hallmarks of multicellularity – the adhesion molecules and adherens junctions are retained in dicyemids and were demonstrated in these organisms earlier by TEM (Furuya et al., 1997). The universal metazoan proteins *Integrin* alpha and *Integrin* beta are detected in dicyemids in single copies; immunoglobulin domain is present in 6 sequences and *Cadherin* in 18 copies.

### Axon Guidance Molecules and Their Receptors

The simplicity of the nervous system in Orthonectida is associated with a reduction of genes encoding components of axon guidance and synapse formation (Mikhailov et al., 2016). Dicyemids are presumably entirely deprived of the nervous system and follow the same trend of gene loss. Both animal groups lack genes encoding semaphorins, important neuronal pathfinding signaling molecules, and their receptors (plexins). Genes potentially involved in the nervous system development, such as Netrin, Ephrins, and Ephrin receptors are present in Orthonectida but were not identified in Dicyemids. Interestingly, the fasciclin domain (PF02469) is absent in the genome of *I. line*i, but we found its three orthologs in *Dicyema*. Fasciclin (FAS1 domain) is a cell adhesion domain found in neural cell adhesion molecules involved in axonal guidance in insects (Grenningloh and Goodman, 1992).

### Peroxisome

The proteins and Pfam domains specific to peroxisome organelles, found in most metazoans, are absent from the dicyemid data. The peroxisomal proteins PEX3, PEX10, PEX12, and PEX19, mandatory for peroxisome function are apparently missing. Failure to detect these genes unequivocally suggests the absence of the organelle. Eight Pfam domains (PF01756, PF04088, PF04614, PF04882, PF05648, PF07163, PF09262, and PF12634) linked to peroxisome in the GO database^1^ were not detected in *Dicyema* spp. In this respect, dicyemids are similar to Orthonectida and parasitic flatworms (Tsai et al., 2013).

### Phylogenetic Analyses

To clarify the relationships of the two mesozoan groups, Orthonectida and Dicyemida, we used the sequenced transcriptomes of two unidentified species of *Dicyema*. We included the gene predictions of the orthonectid *I. linei* (Mikhailov et al., 2016) in the set of orthologous genes based on the dataset published by Struck et al. (2014). Given the high uncertainty in phylogenetic affinities of orthonectids and dicyemids, we extended taxonomic sampling by adding 30 spiralian taxa from available transcriptomes (see section “Materials and Methods”). Although the data broadly covers the spiralian diversity, several taxonomic groups are still missing or underrepresented in the complement of sequenced genes. To minimize missing data, we merged closely related species within several operational taxonomic units (OTUs) and produced the final matrix with 73 OTUs (69 OTUs for spiralian species) and 87,610 aa positions from 452 individual protein alignments. The proportion of missing data in the concatenated alignment is 40%.

**FIGURE 10:**
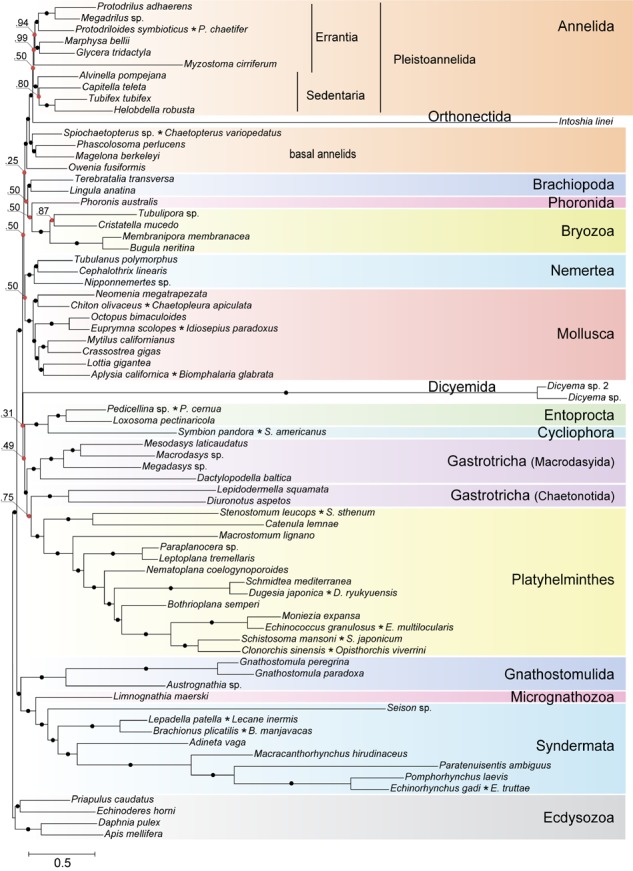
Bayesian tree of Spiralia/Lophotrochozoa with the inclusion of Mesozoa. The consensus topology was constructed from four chains of a PhyloBayes run with the CAT + GTR + Γ4 evolutionary model. Nodes with posterior probabilities below 1.0 are marked with red dots, with those of 1.0 – with black dots. Chimeric operational taxonomic units include names of merged species signed with an asterisk. The tree is rooted with four ecdysozoan lineages.

Highly divergent sequences of mesozoans pose a formidable challenge for inference methods due to the confounding effect of long branched taxa on phylogenetic reconstructions. A recognized approach to tackle the long branch attraction (LBA) problem is to use a site-heterogeneous model of sequence evolution (Rodríguez-Ezpeleta et al., 2007). In the Bayesian tree constructed with PhyloBayes (Lartillot et al., 2013) under the site-heterogeneous CAT-GTR model, the dicyemid and orthonectid lineages form the longest branches, yet they do not group thus contradicting monophyly of the Mesozoa (Figure 10). We recovered the position of the orthonectid *Intoshia* within annelids with the posterior probability of 1.0. Specifically, the orthonectid forms a branch of the Pleistoannelida that comprises the annelid groups Errantia and Sedentaria (Weigert et al., 2014), while *Owenia*, *Magelona*, Chaetopteridae, and *Phascolosoma* (Sipuncula) occupy more basal positions in the annelid subtree.

**FIGURE 11:**
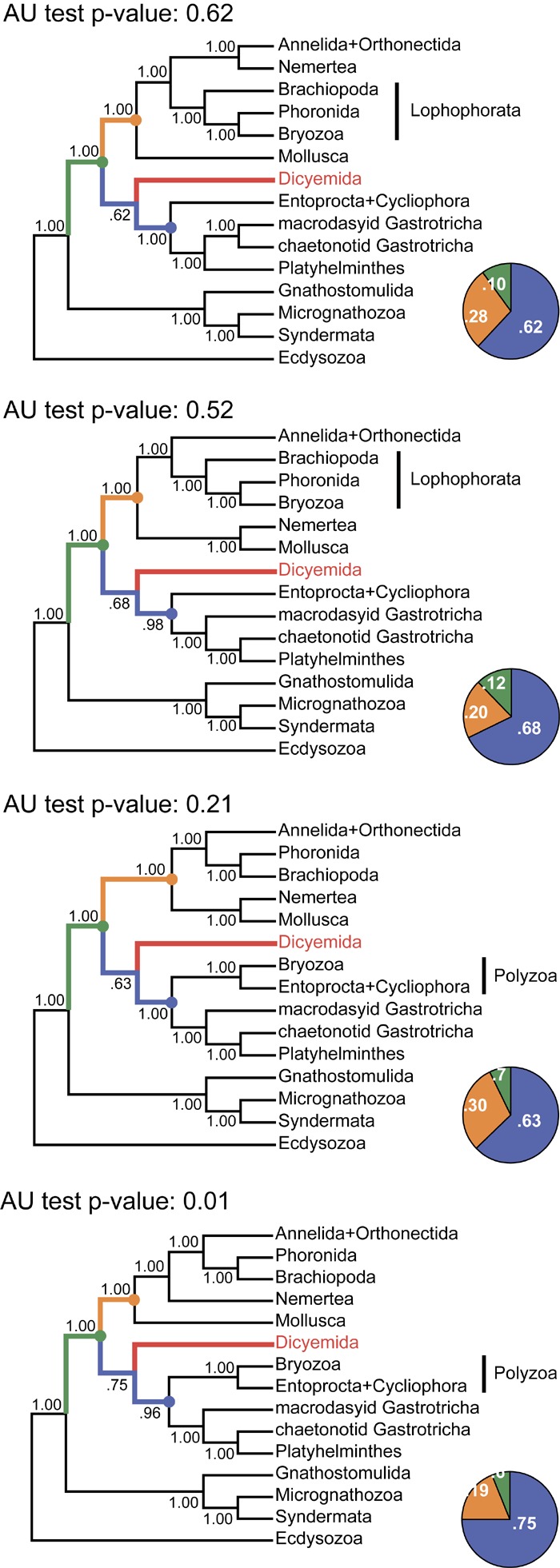
Tree topologies in the four chains of the PhyloBayes run. Each panel summarizes the topology obtained in a single chain of the analysis. The monophyly of almost all clades and all major spiralian phyla receives posterior probability of 1.0 in each chain (even if they differ between the chains). In contrast, the position of the dicyemid lineage receives moderate support in each chain. The pie charts reflect the portion of trees where the dicyemid lineage occupies one of the three observed positions in the cunsensus (represented with color). Topologies in each chain were compared with the approximately unbiased (AU) test using the “sitelogl” option of the PhyloBayes; AU test *p*-values are shown above each topology.

The same analysis placed the dicyemid lineage near the base of a group uniting the Rouphozoa (Platyhelminthes, Gastrotricha) and Entoprocta + Cycliophora. However, the position of dicyemids in Bayesian inference is unstable. In about one-third of trees dicyemids were recovered as a sister group to the clade uniting Annelida, Nemertea, Lophophorata (Brachiopoda + Phoronida + Bryozoa), and Mollusca. In about 10% of trees the dicyemids branch off at the base of this group plus (Platyhelminthes + Gastrotricha) plus (Entoprocta + Cycliophora) (Figure 11, green branch). The grouping of *Intoshia linei* and Pleistoannelida has been observed in all summed trees. However, the exact position of the orthonectids relative to pleistoannelids is less certain in our analyses. The basal placement of the orthonectids is observed in 50% of trees, and the orthonectids were recovered as a sister group of Sedentaria or Errantia in 38 and 11% of trees, respectively.

The consensus Bayesian tree was obtained from four independent chains. The majority of bipartitions are shared across chains, but convergence on a single topology was not observed. Topologies in each chain uniquely reflect the concurrent hypotheses of spiralian relationships (Kocot, 2016; Kocot et al., 2017). All four chains of our Bayesian analysis are consistent in several major assemblages, including the Rouphozoa (Platyhelminthes, Gastrotricha), Gnathifera (Gnathostomulida, Micrognathozoa, and Syndermata), the sister relationship of Rouphozoa and Entoprocta + Cycliophora, and the basal position of Gnathifera relative to other spiralians. Importantly, in all topologies, the orthonectid is nested within the Annelida, and the dicyemid lineage is inferred sister to the assemblage of Platyhelminthes, Gastrotricha, Entoprocta, and Cycliophora (to the inclusion of Bryozoa in some topologies, Figure 11).

Alternative groupings obtained in our analysis include the Lophophorata (Brachiopoda, Phoronida, and Bryozoa) versus Polyzoa (Entoprocta, Cycliophora, and Bryozoa), and Vermizoa (Annelida, Nemertea) versus Nemertea + Mollusca (Figure 11). A comparison of topologies across chains based on site-wise likelihoods computed with PhyloBayes (the “sitelogl” option of the PhyloBayes readpb_mpi) under the CAT-GTR model and the approximately unbiased (AU) test (Shimodaira, 2002) show that the difference in likelihoods of topologies in chains 1–3 is not significant, but the topology likelihood in chain 4 is significantly lower (*p*-value = 0.01). Chain 2 (Figure 11) converges on a topology identical to the consensus four-chains topology (Figure 10) but its likelihood is lower than in chain 1 (non-significantly). Excluding chain 4 that failed the AU test and constructing the consensus with the three remaining chains does not affect the topology itself but only node supports due to eliminating the effects of non-monophyletic Lophophorata in chain 4 (Supplementary Figure S3).

The best scoring topology supports the monophyletic Lophophorata, the grouping of Annelida and Nemertea, and also the monophyly of macrodasyid and chaetonotid gastrotrichs, which frequently find themselves separate in our analyses (Figure 10). Maximum likelihood (ML) analyses of the same dataset with RAxML (Stamatakis, 2014) and IQ-TREE (Nguyen et al., 2015) produce a different view on the phylogeny of Mesozoa. The dicyemids and the orthonectid form a monophyletic group in ML trees with maximal support. In the RAxML analysis under the GTR model the monophyletic Mesozoa branch off with chaetonotid gastrotrichs, similarly to the result obtained in the recent phylogenomic analysis (Lu et al., 2017), but the support of the group is minimal (Supplementary Figure S4). In the IQ-TREE run under the C60 profile mixture model the monophyletic Mesozoa are found at the base of the Rouphozoa, again with weak support (64% of ultrafast bootstrap replicates) (see Supplementary Figure S5).

Although ML analyses show disagreement with the result of Bayesian inference, modeling of site-heterogeneity by the IQ-TREE profile mixture model does shed light on some spurious cases in spiralian relationships. The divergent annelid *Myzostoma* is correctly grouped with other annelids in the IQ-TREE analysis, in contrast with the RAxML tree where it forms a clade with long branches of the Rouphozoa, Gnathifera, and Mesozoa. The clustering of Rouphozoa and Gnathifera referred to as the Platyzoa, receives maximal support in the RAxML analysis but was previously shown to be artefactual (Struck et al., 2014; Laumer et al., 2015). This grouping is not inferred by both the IQ-TREE and Bayesian analyses. In contrast with IQ-TREE and PhyloBayes, the RAxML tree supports monophyletic Polyzoa, a group uniting Entoprocta, Cycliophora, and Bryozoa, which was also suggested to be erroneous and caused by the compositional bias in amino acid sequences (Nesnidal et al., 2013).

To test for expected LBA effects, particularly to exclude the possibility of the orthonectid being attracted to annelids by the divergent *Myzostoma*, we conducted additional analyses excluding each of the long branched lineages. Additional datasets were generated by removing *Myzostoma*, *Myzostoma* and both dicyemids, *Myzostoma* and *Intoshia*. Bayesian analyses of additional datasets recovered placement of *Intoshia* within annelids in the absence of *Myzostoma* (Supplementary Figures S6, S7). The position of dicyemids is also unaffected by the exclusion of other long-branched taxa – the dicyemids occupy a basal position within the Lophotrochozoa after the divergence of Gnatifera in all analyses of the additional datasets (Supplementary Figures S6, S8).

We also tested our dataset for the effects of compositional heterogeneity by discarding highly heterogeneous alignments and utilizing the data recoding approach (Susko and Roger, 2007). Bayesian inference with a concatenate of 150 protein alignments retained after discarding highly compositionally heterogeneous alignments from the original dataset recovers the same groupings of the mesozoan taxa as the analysis of the full dataset. The orthonectid is nested within the annelid clade (1.0 posterior probability) and the dicyemids branch off at the base of the Rouphozoa + Entoprocta + Cycliophora clade with weak support (0.46 posterior probability) (Supplementary Figure S9). Similarly, inference with the Dayhoff-recoded alignment groups the orthonectid with annelids, while leaving the position of the dicyemids uncertain within the Lophotrochozoa (Supplementary Figure S10). Remarkably, the PhyloBayes run with recoded data shows adequate convergence between chains (*maxdiff* = 0.17) and infers the monophyletic Gastrotricha. Several conventional groupings, such as the Rouphozoa, are not recovered. Consistent with the proposed artefactual nature of the grouping of Bryozoa and Entoprocta due to compositional heterogeneity (Nesnidal et al., 2013), both test datasets support the monophyletic Lophophorata, whereas the alternative Polyzoa was frequently observed for the complete and non-recoded data. The lack of support for monophyletic Rouphozoa in the analysis with the recoded dataset was similarly obtained in a recent study of the spiralian phylogeny, which aimed at counteracting the impact of compositional heterogeneity (Marlétaz et al., 2019).

**FIGURE 12:**
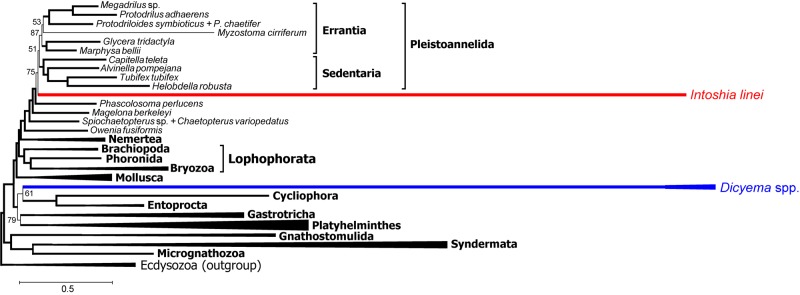
PhyloBayes topology for proteins with the strong annelid signal (concatenate of 111 protein alignments that possess at least 3% positions with *q* ½, 18686 alignment positions in total, CAT + GTR + Γ4 model, 50,000 chain steps, 50% burn-in). Only posterior probability values less than 0.95 are shown. Convergence value of *maxdiff* = 0.15.

Schiffer et al. (2018) selected proteins that support annelid monophyly as an approach to verify the orthonectid position. We also selected 111 protein alignments that contain the annelid signal but with a different method, and used those for Bayesian inference with the PhyloBayes program. In contrast to other Bayesian runs, the consensus presents a stable topology (*maxdiff* value 0.15). In this tree, the orthonectid *I. linei* stabilizes inside the annelids [posterior probability (PP) 1.0], whereas the species of *Dicyema* are not attracted to annelids (Figure 12). The position of *Dicyema* remains uncertain within the lineage of long-branched taxa (Platyhelminthes, Gastrotricha, Entoprocta, Cycliophora). Lophophorata and Gastrotricha are reconstructed with PP 1.0 (as in case of the Dayhoff-recoded dataset mentioned above and the non-recoded full dataset in chain 1 that reaches the highest likelihood). Bayesian topologies obtained in chain 1 (Figure 11) and both the sub-sampled datasets of 111 proteins with the annelid signal and the 150 proteins with low compositional heterogeneity (Supplementary Figure S9) reconstruct the sister relationship of annelids with nemertines. Marlétaz et al. (2019) also report the grouping of annelids and nemertines, with the inclusion of Platyhelminthes.

The lack of convergence in most PhyloBayes analyses precludes strong assertions regarding problematic areas of the spiralian tree. Nevertheless, some clades are reconstructed consistently. We do not observe monophyly of the Mesozoa in any of the chains, in contrast to the recent study by Lu et al. (2017) and in agreement with the evidence from Schiffer et al. (2018), nor do we observe their direct relations with Platyhelminthes.

The orthonectid *I. linei* occupies a stable position within the annelid part of the tree. Its placement is among the major conflicts between ML and Bayesian topologies, which likely indicates the impact of a more complex CAT-GTR model in the presence of long branches of highly divergent lineages like orthonectids and dicyemids. Noteworthy, polyphyletic Mesozoa and the proposed affinity of orthonectids to annelids was also recovered in Schiffer et al. (2018) in Bayesian analyses of a dataset with the less extensive representation of the lophotrochozoan diversity.

The orthonectids share with annelids certain morphological features: the presence of microvillar cuticle, metameric muscles, gonochory (Slyusarev, 2008), and the dorsal ganglion in adult specimens (Slyusarev and Starunov, 2015). Cases of dramatic morphological reduction in annelids are known in archiannelids (Andrade et al., 2015) and lobatocerebrids (Laumer et al., 2015), and especially in dwarf males of the echiurid *Bonnelia*, dinophidid *Dinophilus gyrociliatus*, spionid *Scolelepis laonicola* (Vortsepneva et al., 2008), and siboglinids (Worsaae and Rouse, 2010). Adaptations of orthonectids that had led to the complete loss of coelomic cavity, gonad wall, chaetae, gastral system, nephridia, trochophore larva, and spiral cleavage further demonstrate the extent of morphological regress in the evolution of annelids.

The dicyemid lineage in our analyses exhibits affinity to the Rouphozoa clade, in congruence with Lu et al. (2017), with the intercalation of Entoprocta or Polyzoa, which were not included in analyses by Lu et al. (2017). Schiffer et al. (2018) report an uncertain position of dicyemids at the base of the Lophotrochozoa. We did not recover the position of the dicyemids as part of the Platyhelminthes. A previous analysis of innexin genes (Suzuki et al., 2010) also rejects the kinship of the dicyemids and Platyhelminthes. Furthermore, rhabditophoran platyhelminths are known to possess a unique non-standard mitochondrial genetic code, which was shown to be not the case for the studied mesozoans (Telford et al., 2000; Schiffer et al., 2018). Dicyemids were placed within Spiralia in various taxonomic contexts in molecular phylogenetic studies (Pawlowski et al., 1996; Petrov et al., 2010; Suzuki et al., 2010; Lu et al., 2017) but the interpretation of their body plan remains enigmatic. Being among the simplest known bilaterians, they yet possess multiciliated epithelia, which is not a primitive trait and suggests secondary evolutionary regress, and do not display evident synapomorphies with other animal phyla. Dicyemids might represent a relict lineage of lophotrochozoan animals with no direct relatives that had survived to the present days.

### Conclusion

We confirm that orthonectids are extremely simplified annelids and do not form a monophyletic group with dicyemids. Mesozoa is a polyphyletic taxon. Dramatic simplification of their body plan, as well as the metagenetic life cycle, evolved independently in the two lineages. Many conserved bilaterian genes are absent in the genomes of Dicyemida and Orthonectida. At the same time, the pattern of their loss and presence is different, which supports the conclusion that these animal groups are not close relatives and have simplified independently. Analyses of genes related to the basement membrane, neuronal and muscular systems expose the extreme simplicity of dicyemids. Intriguingly, dicyemids lack muscle cells and the genetic factors of muscle cell differentiation but possess the troponin complex specific for striated muscles. Taken together with detection of a relatively big set of nicotinic acetylcholine receptors often associated with neuromuscular signaling and the presence of voltage-gated ion channels, this fact urges reevaluation of the traditional view that dicyemids completely lost the neuro-muscular system. Appealing is to experimentally check if some contractility and movements could be induced in dicyemids by signal molecules such as acetylcholine or glutamate, and for the presence of electrical excitability in the form of propagated calcium action potential in their cells. Small circular extrachromosomal molecules are present in total DNA extracts of dicyemids. Mitochondrial rRNA, tRNA, protein-coding genes and pseudogenes are located on circular molecules. There are short nucleotide sequence motifs confined specifically to circular DNAs in *Dicyema* sp.

## Materials and Methods

### Biological Material, Genome and Transcriptome Sequencing

The original live material on *Dicyema* sp. 1 was collected at the Vostok marine biological station of the Institute for Marine Biology of the Russian Academy of Sciences (the Vostok Bay of the Sea of Japan, Vladivostok, Russia) from dissected kidneys of the giant Pacific octopus *E. dofleini*. Live dicyemids were rinsed individually in filtered marine water and fixed in the RNAlater stabilization solution (Ambion). Total DNA was isolated from tissue samples by Diatom DNA Prep (IsoGene). The sequencing of dicyemid genomic data was performed with an Illumina HiSeq2000 system, generating 140 million paired-end reads.

Total RNA was isolated by TRIzol kit (Invitrogen) and further used for ds cDNA synthesis using the SMART approach (Zhu et al., 2001). SMART-prepared amplified cDNA was then normalized using the DSN normalization method (Zhulidov et al., 2004). Normalization included cDNA denaturation/reassociation, treatment by the duplex-specific nuclease (Shagin et al., 2002), and PCR amplification of the normalized fraction (8 PCR cycles: 95°C for 7 s; 65°C for 20 s; 72°C for 3 min). Normalized cDNA libraries were sequenced using the Roche 454 sequencing technology, producing about 480,000 reads with an average length of 444.6 bases.

Specimens of *Dicyema* sp. 2 was collected at the Friday Harbor Laboratories (Friday Harbor, WA, United States) from circulatory system and kidneys of the octopus *E. dofleini*. All individual animals were washed 3–5 times in 0.2 μm filtered seawater. Then RNA was extracted from individual animals and processed as described elsewhere (Moroz and Kohn, 2013) for Illumina HiSeq 2000 sequencing.

The sequences are deposited in the NCBI: BioProject PRJNA527259 (*Dicyema* sp. 1) and SRA SRP021079 (*Dicyema* sp. 2).

### Assembly and Filtering of Dicyemid Sequences

The reads obtained from the DNA library for *Dicyema* sp. 1 were trimmed for adapters with Trimmomatic (Bolger et al., 2014) and assembled by SPAdes (Nurk et al., 2013) using *k*-mer values of 21, 33, 55, and 77. We also performed genome assembly with the Newbler GS De Novo Assembler software (v. 2.9) (using 1/10 of all reads) as a control to our method of circular contigs identification. Gene prediction was performed with Augustus (Stanke and Waack, 2003) after constructing a training set of 200 dicyemid sequences identified in the genomic assembly. The predicted genes were queried against the InterPro database (Finn et al., 2017) with InterProScan (Jones et al., 2014) and genes with InterPro hits were screened for cephalopod sequences with BLAST (Altschul et al., 1997) searches against the NCBI *nr* database. Predictions producing best hit with cephalopod sequences were discarded from the gene set. Completeness estimates were performed with BUSCO (Waterhouse et al., 2017) using the eukaryota_odb9 ortholog set (Zdobnov et al., 2017). HMMER (Eddy, 2011) searches were carried out with Pfam (Finn et al., 2016) Homeodomain (PF00046) and Homeobox_KN (PF05920) profiles to identify homeobox transcription factors in the data. Phylogeny reconstructions for homeobox sequences were performed with IQ-TREE (Nguyen et al., 2015) using the LG + C20 + G4 model of sequence evolution or with PhyloBayes (Lartillot et al., 2013) using the LG +CAT + G4 model.

The reads obtained from the cDNA library for *Dicyema* sp. 1 were trimmed for adapters, non-coding RNA, low-quality and low-complexity sequences with the SeqClean software (Dana-Farber Cancer Institute^2^), and about 430,000 reads were retained. Data was further assembled with the original 454 Newbler GS De Novo Assembler software (v. 2.9) utilizing flowgram quality data and settings that maximize contig overlap. The “-urt” option was invoked to improve contigging in low depth portions of the assembly. Fusions of transcripts that can potentially occur with low-depth assembly extensions in densely packed genomes are subsequently eliminated in our experimental design by alignment filtering at the supermatrix construction step. The obtained assembly contained 19,641,638 bases, and 22,082 isotigs of average size 889 bases, N50 size of 1,081, and the largest isotig size of 9,199. Protein coding regions were predicted using TransDecoder (Haas et al., 2013) with settings to maximize the sensitivity of capturing ORFs regardless of the predicted coding likelihood score by accounting for homology to known proteins in the Pfam (Finn et al., 2014) and UniProtKB/Swiss-Prot (UniProt Consortium, 2015) curated databases. Coding region prediction with TransDecoder was set to the minimal predicted protein length of 80 aa. The predicted proteome contained 15,227 unique coding regions.

The second dicyemid transcriptome sequenced using the Illumina platform was assembled with Trinity (Grabherr et al., 2011). Before assembly the reads were processed with the SeqClean software, and the prediction of coding regions was performed by TransDecoder, similarly to the transcriptome of the first dicyemid.

The transcriptomes of dicyemids were derived from samples contaminated with their cephalopod host. Therefore, we paid special attention to avoid mixing dicyemid and cephalopod sequences in the phylogenetic analysis. The transcriptomes of dicyemids were first screened for cephalopod sequences by performing BLAST (Altschul et al., 1997) searches against the NCBI RefSeq database (O’Leary et al., 2016). Two dicyemid transcriptomes were processed independently. In the first step of decontamination we filtered out proteins having best hit in RefSeq belonging to prokaryotes (and having at least 50% identity). This lead to rejection of only 35 proteins for *Dicyema* sp. 1 and 363 proteins for *Dicyema* sp. 2. In the second step we removed all dubious proteins if their local alignment score with any cephalopod protein higher than in all the other considered species (with the same query protein). Sequences with best hits to cephalopods were discarded from the transcriptomes if the sequence identity exceeded 70%. For the third filtering step we queried the proteins of *O. bimaculoides* combined with several transcriptomes of *Octopus vulgaris* (NCBI BioProject PRJNA79361 and Sequence Read Archive entries SRR331946, SRR1507221) against a custom database containing 9 metazoan proteomes (4 molluscs, 2 annelids, a brachiopod *Lingula anatina*, an ecdysozoan *Limulus polyphemus*, and a deuterostome *Danio rerio*) and the dicyemid transcriptome, and inspected dicyemid sequences that produced hits with the highest match to the cephalopod queries among the 10 metazoans. All dubious sequences (hits with at least 80% identity) captured by this method were discarded from the dicyemid transcriptomes as potential cephalopod contamination.

### Search for “Circular” Contigs, Signals, and Mitochondrial Sequences

The contigs constructed from shotgun fragments display special characteristics emerging from the genome assembly algorithms based on De Bruijn graph of *k*-mers. This approach results in “circular” contigs starting and ending with the same *k*-mer. After assembly, terminal repeats equal in length to the *k*-mer were cut off. Contigs analyzed in sections “Circular Contigs in Genomic Assembly of *Dicyema* sp.” and “Mitochondrial DNA of *Dicyema* sp.,” and NCBI submission data have been cleaned off the terminal repeats. In this study, a contig was considered “circular” if it had terminal direct repeats ≥ 77 nt in length (k77). The length distribution of contigs assembled by different methods (Newbler and SPAdes) was compared with the two-sample Kolmogorov–Smirnov test implemented in the SciPy package in Python 3. Here the null hypothesis is that contig lengths come from the same distribution. High *p*-values in this case reflect high probabilities of this hypothesis. Low complexity regions were detected with the DUST algorithm from the MEME Suite (Bailey and Elkan, 1994) with standard settings. MEME and ChIPMunk (Kulakovskiy et al., 2010) tools with the default parameters were applied to the task of finding specific motifs. The reverse lookup for the signal presence was done via FIMO (from the MEME Suite) with the *p*-value threshold of 10^−4^. Moreover, highly conserved elements of circles in dicyemids were found utilizing the technique borrowed from (Rubanov et al., 2016). The method identifies highly conserved DNA elements on the base of the identification of dense subgraphs in a specially built multipartite graph (whose parts correspond to genomes). Specifically, the algorithm does not rely on genome alignments, no pre-identified perfectly conserved elements; instead, it performs a fast search for pairs of words (in different genomes) of maximum length with the difference below the specified edit distance. Such pair defines an edge whose weight equals the maximum (or total) length of words assigned to its ends. The graph composed of these edges is then compacted by merging some of its edges and vertices. The dense subgraphs are identified by a cellular automaton-like algorithm; each subgraph defines a cluster composed of similar inextensible words from different genomes (Rubanov et al., 2016).

HMMER3 package (Eddy, 2011) along with the Pfam-A database were used to find the circles containing protein-coding sequences, whereas an additional verification step was performed in BLAST. The search itself was conducted through the database composed of six-frame translated circular sequences. The search for genes coding for mitochondrial proteins was conducted with BLAST using mitochondrial protein-coding gene sequences from flatworms as queries, MITOS (Bernt et al., 2013) and HMMER3 using HMM profiles from the Pfam-A database. Mitochondrial rrnS genes in dicyemids are highly diverged and poorly detected with BLAST. Their detection was conducted with HMMER3 with HMM profiles preliminarily generated from the set of 140 rrnS alignments from other organisms (140 species of bilaterians, cnidarians, and placozoans). All findings were verified using *blastp* or *blastn* with *nr* NCBI database. It was proposed that the dicyemid small mitochondrial circular DNA molecules are generated from the usual long multigene mitochondrial DNA (Awata et al., 2006). If such long mtDNA exists together with mitochondrial mini-circles we can expect the cases when one read from the sequencing library corresponds to a particular mitochondrial mini-circle while its pair read maps elsewhere. *Blastn* with minimal word size was used to map raw paired end reads to circular contigs coding for mitochondrial genes to search for hypothetical high-molecular-weight mtDNA. Reads pair analysis was conducted after that in order to find the reads whose pair does not map to the initial circular contig. Mitochondrial tRNA secondary structures were predicted using the MiTFi program (Jühling et al., 2012).

### Taxonomic Expansion of Alignments

The starting set of orthologous genes used in this work is based on a dataset for phylogenetic reconstructions within Spiralia assembled by Struck et al. (2014) that was later expanded with sequences of orthonectid *I. linei* (Mikhailov et al., 2016). The base set of orthologs contained 469 alignments with a total of 62 spiralian species and four ecdysozoan species. To extend the taxonomic sampling of Spiralia and minimize the missing data in the dataset we obtained predicted proteins from several genomic projects accessible through public databases and collected transcriptomic data from the NCBI Sequence Read Archive. The annotations for the genomes of *Clonorchis sinensis*, *Echinococcus granulosus*, *L. anatina*, *O. bimaculoides*, *Priapulus caudatus* were obtained from the GenBank database, and the proteins of *Adineta vaga* were obtained from the Genoscope database. The NCBI Sequence Read Archive was used to extract raw sequence data of another 31 spiralian species (see Supplementary Table S1).

The assemblies of the SRA transcriptome data were performed with Trinity (Grabherr et al., 2011) after cleaning the reads with SeqClean (Dana-Farber Cancer Institute^3^) from adapter sequences using the UniVec_Core database^4^ and filtering ribosomal RNA sequences using a database of eukaryotic rRNAs. The prediction of proteins in the assembled transcripts was performed with TransDecoder (Haas et al., 2013), which was assisted with searches against the Pfam (Finn et al., 2014) and UniProtKB/Swiss-Prot (UniProt Consortium, 2015) databases.

The addition of proteins from the newly assembled data to orthologous groups featured in the base set of alignments was performed using the procedure for mapping genes to existing orthologous groups (Fischer et al., 2011) of the OrthoMCL database (Chen et al., 2006). The genes from the initial dataset and novel transcriptomic and genomic data were assigned to orthologous groups of OrthoMCL-DB, and the genes within the same orthologous group were extracted and aligned together using MUSCLE (Edgar, 2004). When more than one sequence per organism was assigned to the same group of orthologs, only the sequence scoring highest against the orthologous group in the initial dataset was selected for the alignment.

### Phylogenetic Analyses

The concatenation of individual gene alignments was performed with Scafos (Roure et al., 2007) using the option to construct chimeric sequences for several closely related taxa. The following 15 chimeric taxa were constructed for the analysis: *Aplysia californica* + *Biomphalaria glabrata*, *Brachionus plicatilis* + *B. manjavacas*, *Chiton olivaceus* + *Chaetopleura apiculata*, *Clonorchis sinensis* + *Opisthorchis viverrini*, *Dugesia japonica* + *Dugesia ryukyuensis*, *Echinococcus granulosus* + *Echinococcus multilocularis*, *Echinorhynchus gadi* + *Echinorhynchus truttae*, *Euprymna scolopes* + *Idiosepius paradoxus*, *Lepadella patella* + *Lecane inermis*, *Pedicellina* sp. + *P. cernua*, *Protodrilloides symbioticus* + *P. chaetifer*, *Schistosoma mansoni* + *S. japonicum*, *Spiochaetopterus* sp. + *Chaetopterus variopedatus*, *Stenostomum leucops* + *Stenostomum sthenum*, *Symbion pandora* + *S. americanus*. Another ten species that were present in the starting set of alignments were removed due to poor representation in the final alignment: *Alcyonidium diaphanum*, *Fasciola gigantica*, *Flustra foliacea*, *Lumbricus rubellus*, *Philodina roseola*, *Rotatoria rotatoria*, *Spirometra erinacei*, *Stylochoplana maculata*, *Taenia solium*, *Turbanella ambronensis*. The final number of operational taxonomic units featured in the analysis is 73. Before concatenation, the alignments were trimmed with TrimAl (Capella-Gutierrez et al., 2009) to remove poorly aligned regions. The trimming was performed with a gap threshold of 0.5 and a similarity threshold of 0.001. After the removal of invariant positions, the length of the concatenated alignment totaled 87,610 positions, with 40% missing data. Compositional heterogeneity in the alignment partitions (i.e., individual protein alignments after masking) was evaluated using the relative composition frequency variability (RCFV) metric (Zhong et al., 2011). The RCFV values were calculated using BaCoCa (Kuck and Struck, 2014). The low compositional heterogeneity dataset was generated by discarding 302 partitions (referred to in the paper simply as protein alignments) with RCFV value exceeding 0.115.

The phylogenetic reconstructions were performed with PhyloBayes-MPI 1.7 (Lartillot et al., 2013), RAxML (Stamatakis, 2014), and IQ-TREE (Nguyen et al., 2015). The RAxML analysis was carried out utilizing the complete analysis function (-f a) with 150 rapid bootstrap replicates and the PROTCATGTR model of evolution. The IQ-TREE analysis was performed using the LG + C60 + F + G4 evolutionary model, and node support was calculated using the ultrafast bootstrap approximation (Minh et al., 2013) with 1,000 replicates. The Bayesian inference with PhyloBayes was carried out using the CAT + GTR + Γ4 model, and the analyses were run with four chains. For the main dataset, the majority rule consensus tree was reconstructed after 30,000 cycles using one out of ten cycles with a 60% burn-in. PhyloBayes analyses of the additional datasets were conducted similarly to the main dataset; the consensus trees were reconstructed after 5,000 or 15,000 cycles with a 50% burn-in. Analysis of the recoded alignment was performed with PhyloBayes utilizing the recode option and the Dayhoff recoding scheme with six amino acid groups (Dayhoff et al., 1978). The convergence of the chains was assessed by comparing bipartitions using the *pbcomp* utility from the PhyloBayes package.

Comparison of topologies in the four chains of the Bayesian inference of the main dataset was performed using the CONSEL program (Shimodaira and Hasegawa, 2001) and the “sitelogl” option of the PhyloBayes readpb_mpi program. The site-specific marginal log likelihoods were computed for each chain across 10 data points sampled over 2,000 cycles after a 20,000 cycle burn-in.

Alignment partitions (i.e., individual protein alignments after masking) with the strong annelid signal were selected as follows. In a protein alignment we define two sets of sequences – *G*_1_ (ingroup), and *G*_2_ (outgroup). Only alignment positions containing no more than a half of missing data (gaps or X’s) in each of the two sets are considered. For each such position *i*-value *q*(*i*) is determined as the maximum of frequency differences of each amino acid in this position from *G*_1_ and *G*_2_. Missing data is ignored. Maximum *q*(*i*) value is 1 when *G*_1_ consists only of one character, and *G*_2_ does not contain this character. Under any *q*(*i*) ½ there exists a character *a*(*i*) observed in more than a half of taxa from *G*_1_ but much less frequently in *G*_2_ [frequency difference is *q*(*i*) ½]. In the phylogenetic context, when *G*_1_ + *G*_2_ constitute a monophyletic clade, and *G*_1_ is a narrower natural clade, high *q*(*i*) values can be interpreted as presence of a synapomorphy against *G*_2_. Notably, in this analysis *q*(*i*) values are used only to select partitions but not for alignment editing or positions removal. In our case of detecting the annelid signal, *G*_1_ contained all annelids except the orthonectid, and *G*_2_ – all non-annelid taxa except dicyemids in order to obtain *q*(*i*) estimates unbiased with respect to the lineages under study.

## Author Contributions

OZ performed most of the computations, analyzed the data, and drafted the manuscript. KM, YP, SI, OP, and LR performed additional computations, analyzed the data, and wrote the manuscript. LR obtained original RNA-Seq data, assembled the transcriptome of *Dicyema* sp. 1. ML and AP obtained original DNA-Seq data. LM obtained original RNA-Seq data on *Dicyema* sp. 2. VL supervised the computational part of the work. VA designed and supervised the research. All authors read and approved the manuscript.

## Conflict of Interest Statement

The authors declare that the research was conducted in the absence of any commercial or financial relationships that could be construed as a potential conflict of interest.

## Footnotes

1 http://geneontology.org/external2go/pfam2go

2 https://sourceforge.net/projects/seqclean

3 https://sourceforge.net/projects/seqclean

4 http://www.ncbi.nlm.nih.gov/tools/vecscreen/univec/
