# Supplementary material for: Dicyemida and Orthonectida: Two Stories of Body Plan Simplification
Source: Front Genet. 2019 May 24;10:443. doi: 10.3389/fgene.2019.00443 (PMC6543705; doi:10.3389/fgene.2019.00443)
Supplement: Supplementary file 1 [file Data_Sheet_1.pdf]

Frontier in Genetics

doi: 10.3389/fgene.2019.00443

Supplementary material

Dicyemida and Orthonectida: Two stories of body plan simplification

Oleg A. Zverkov[1], Kirill V. Mikhailov[1,2], Sergey V. Isaev[1,3], Leonid Y. Rusin[1,4], Olga V. Popova[2], Maria D. Logacheva[1,2,5], Alexey A. Penin[1,4], Leonid L. Moroz[6], Yuri V. Panchin[1,2], Vassily A. Lyubetsky[1], and Vladimir V. Aleoshin[1,2*]

[1]Institute for Information Transmission Problems, Russian Academy of Sciences, Moscow, 127051, Russian Federation,

[2]Belozersky Institute for Physico-Chemical Biology, Lomonosov Moscow State University, Moscow, Russian Federation,

[3]Faculty of Bioengineering and Bioinformatics, Lomonosov Moscow State University, Moscow, Russian Federation,

[4]Faculty of Biology, Lomonosov Moscow State University, Moscow, Russian Federation,

[5]Skolkovo Institute of Science and Technology, Moscow, Russian Federation,

[6]Department of Neuroscience and McKnight Brain Institute, University of Florida, Gainesville, 32611, USA

Table 1 (for page 17)**.** NCBI Sequence Read Archive accessions of transcriptome libraries used in the phylogenetic analysis

Taxonomic group	Species	Experiment ID	Run ID(s)
**Acanthocephala**	*Echinorhynchus gadi*	SRX1121912	SRR2131254
**Annelida** (Errantia)	*Glycera tridactyla*	SRX516622	SRR1237833,70
	Marphysa bellii	SRX515220	SRR1232833
	Myzostoma cirriferum	SRX516623	SRR1237872
**Annelida** (Protodrilidae)	*Megadrilus* sp.	SRX1026327	SRR2020581
	Protodriloides chaetifer	SRX1023293	SRR2016233
	Protodriloides symbioticus	SRX1116278	SRR2124791,92
	Protodrilus adhaerens	SRX1022008	SRR2014684
**Annelida** (early branches)	*Chaetopterus variopedatus*	SRX512002	SRR1219647
	Magelona berkeleyi	SRX522872	SRR1257638,39
	Owenia fusiformis	SRX512807	SRR1222288
	Spiochaetopterus sp.	SRX513557	SRR1224605
**Brachiopoda**	*Terebratalia transversa*	SRX1015908	SRR2005824
**Bryozoa**	*Membranipora membranacea*	SRX1121923	SRR2131259
**Dicyemida**	*Dicyema* sp.	SRX265465	SRR827581
**Gastrotricha**	*Diuronotus aspetos*	SRX1121926	SRR2131262
	Mesodasys laticaudatus	SRX872416	SRR1797883
**Gnathostomulida**	*Austrognathia* sp.	SRX997150	SRR1976176
**Kamptozoa**	*Loxosoma pectinaricola*	SRX731476	SRR1611559
	Symbion americanus	SRX1122263	SRR2131690
**Micrognathozoa**	*Limnognathia maerski*	SRX1121929	SRR2131287
**Mollusca**	*Chiton olivaceus*	SRX205322	SRR618506
**Nemertea**	*Nipponnemertes* sp.	SRX647389	SRR1508368
**Phoronida**	*Phoronis australis*	SRX1025550	SRR2018856
**Platyhelminthes** (Catenulida)	*Catenula lemnae*	SRX871445	SRR1796434
	Stenostomum leucops	SRX951992	SRR1910423
	Stenostomum sthenum	SRX872404	SRR1801788
**Platyhelminthes** (Trepaxonemata)	*Bothrioplana semperi*	SRX980750	SRR1955240
	Leptoplana tremellaris	SRX872321	SRR1797726
	Nematoplana coelogynoporoides	SRX872398	SRR1797817
**Rotifera**	*Lepadella patella*	SRX997434	SRR1976570
**Sipuncula**	*Phascolosoma perlucens*	SRX755855	SRR1646442

Supplementary Figure S1 (for page 4). Distribution of circular contig lengths in various assemblies. Discrete length distribution is observed in assemblies by SPAdes (based on De Bruijn graphs, *k*-mer size 55 or 77 bases), as well as Newbler (not based on De Bruijn graphs).

Supplementary Figure S2 (for page 5). Predicted secondary structures of putative mitochondrial tRNAs of *Dicyema* sp. Co-locating genes and paralogs are outlined (solid and dashed, respectively).

Supplementary Figure S3 (for page 13). Bayesian tree of the Spiralia/Lophotrochozoa with inclusion of the Mesozoa. By supplementing **Figure 10**, it depicts the consensus of the three chains that passed the AU-test, while **Figure 10** shows the all four-chains consensus (*maxdiff*=1.0).

Supplementary Figure S4 (for page 13). Maximum likelihood reconstruction with RAxML. The tree was obtained under the PROTCATGTR model; support values are based on 150 rapid bootstrap replicates. Values of 100% are not shown, and corresponding nodes are marked with black dots. Chimeric operational taxonomic units include names of merged species signed with an asterisk.

Supplementary Figure S5 (for page 13). Maximum likelihood reconstruction with IQ-TREE. The inference was performed under the LG+C60+F+G4 model; node support was evaluated with ultrafast bootstrap approximation (1000 replicates). Nodes with 100% support marked with black dots.

Supplementary Figure S6 (for page 14). Bayesian inference with the dataset excluding the annelid *Myzostoma cirriferum*. PhyloBayes was run with four chains under the CAT+GTR+Γ4 model. The consensus tree was constructed after 15,000 cycles with 50% burn-in (*maxdiff* =1.0). Only posterior probability values below 1.0 are shown.

Supplementary Figure S7 (for page 14). Bayesian tree with the exclusion of two dicyemids and the annelid *Myzostoma cirriferum*. PhyloBayes was run with four chains under the CAT+GTR+Γ4 model. The consensus tree was constructed after 5,000 cycles with 50% burn-in (*maxdiff*=1.0). Only posterior probability values below 1.0 are shown.

Supplementary Figure S8 (for page 14). Bayesian tree with the exclusion of the orthonectid *Intoshia linei* and the annelid *Myzostoma cirriferum*. PhyloBayes was run with four chains under the CAT+GTR+Γ4 model. The consensus tree was constructed after 5,000 cycles with 50% burn-in (*maxdiff*=1.0). Only posterior probability values below 1.0 are shown.

Supplementary Figure S9 (for page 14). Bayesian inference with the 35K-site dataset excluding protein alignments with high compositional heterogeneity. The inference was carried out under the CAT+GTR+Γ4 model, and the tree was generated after 50,000 cycles with 50% burn-in (*maxdiff*=0.95).

Supplementary Figure S10 (for page 14). Bayesian inference with the Dayhoff-recoded alignment. The inference was carried out under the CAT+GTR+Γ4 model; the tree was generated after 30,000 cycles with 50% burn-in (*maxdiff*=0.17).

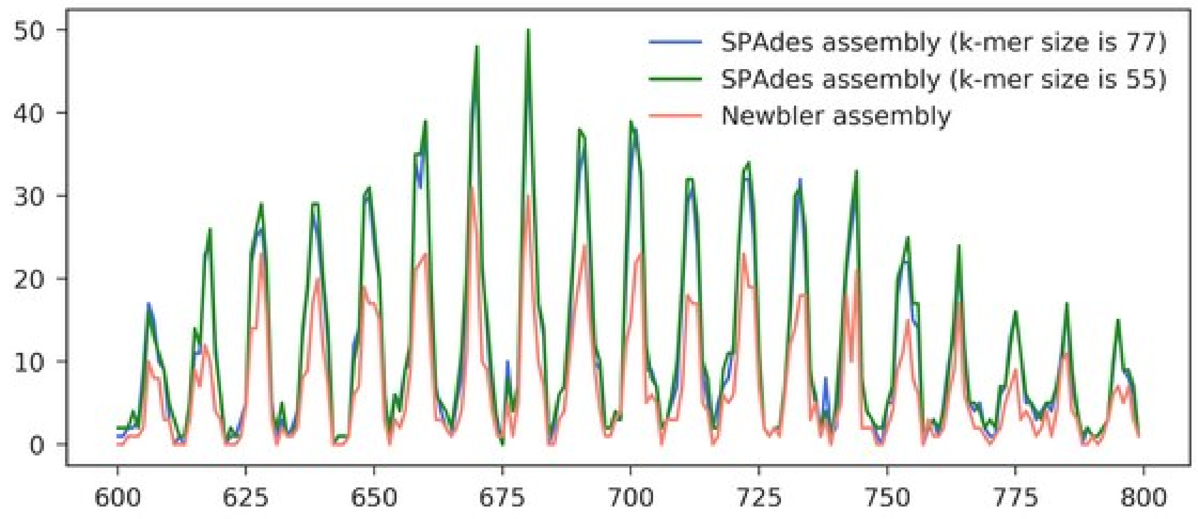

Supplementary Figure S1 (for page 4)**.** Distribution of circular contig lengths in various assemblies. Discrete length distribution is observed in assemblies by SPAdes (based on De Bruijn graphs, *k*-mer size 55 or 77 bases), as well as Newbler (not based on De Bruijn graphs).

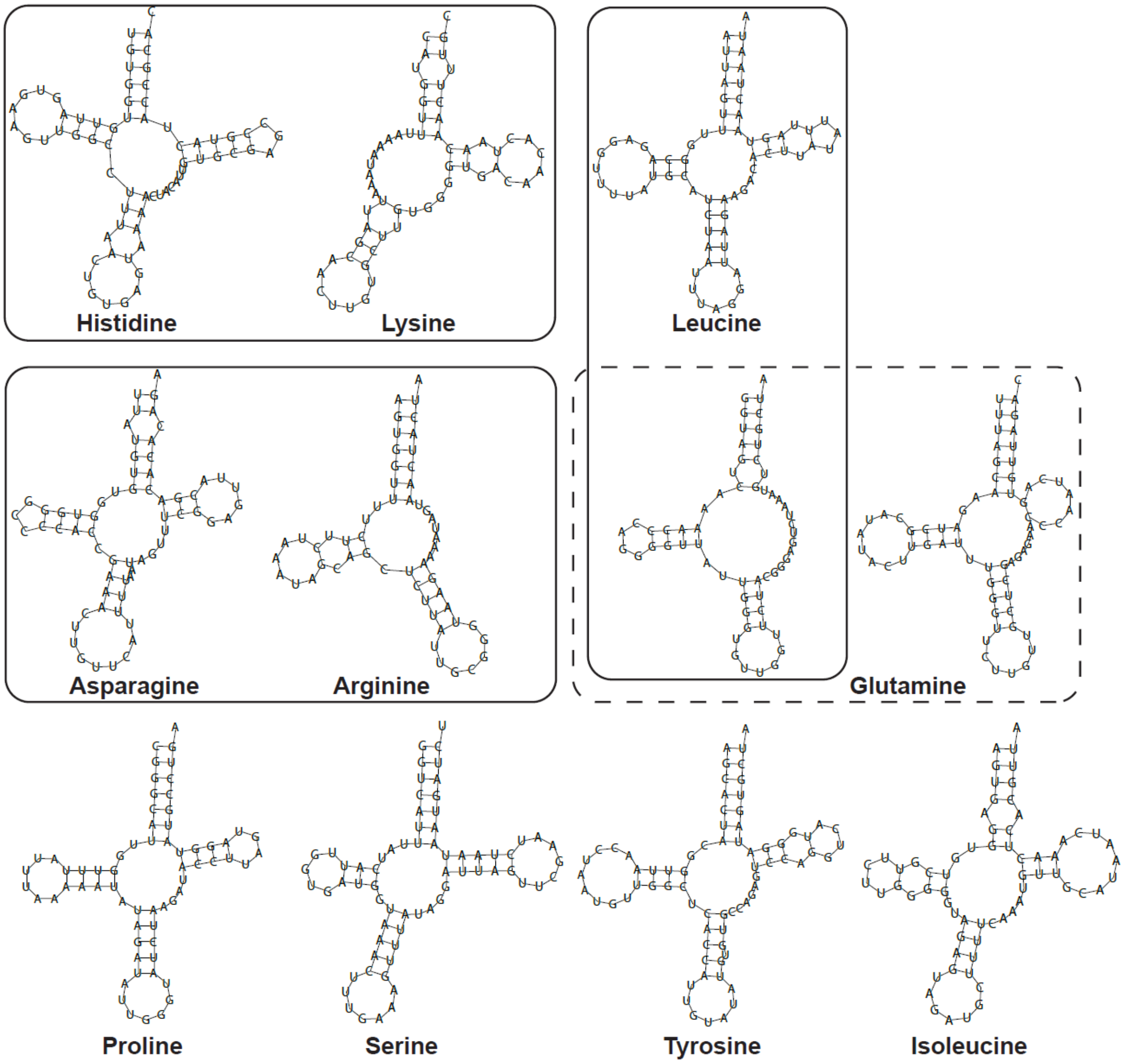

Supplementary Figure S2 (for page 5)**.** Predicted secondary structures of putative mitochondrial tRNAs of *Dicyema* sp. Co-locating genes and paralogs are outlined (solid and dashed, respectively).

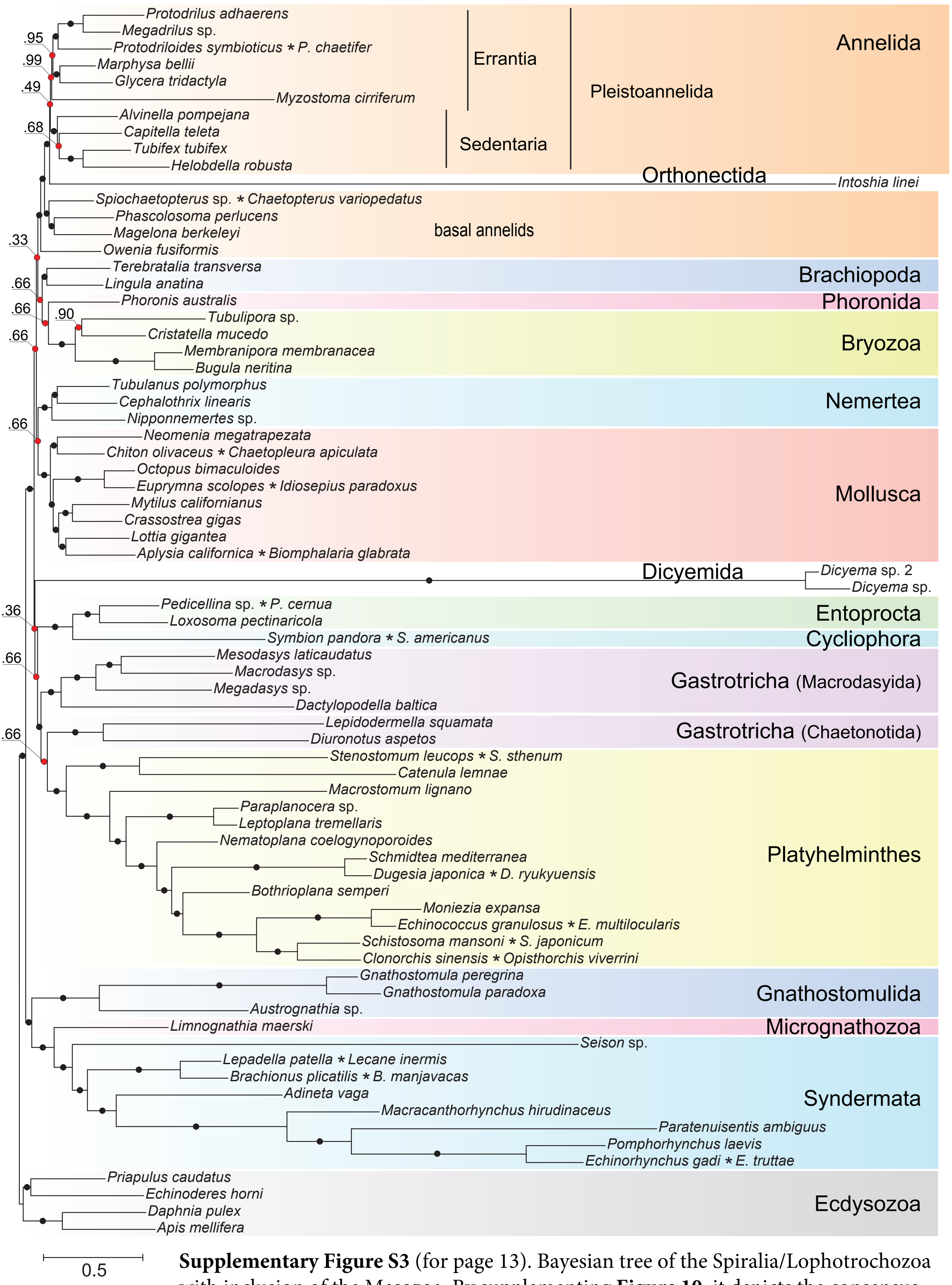

Supplementary Figure S3 (for page 13). Bayesian tree of the Spiralia/Lophotrochozoa with inclusion of the Mesozoa. By supplementing **Figure 10**, it depicts the consensus of the three chains that passed the AU-test, while **Figure 10** shows the all four-chains consensus (*maxdiff*=1.0).

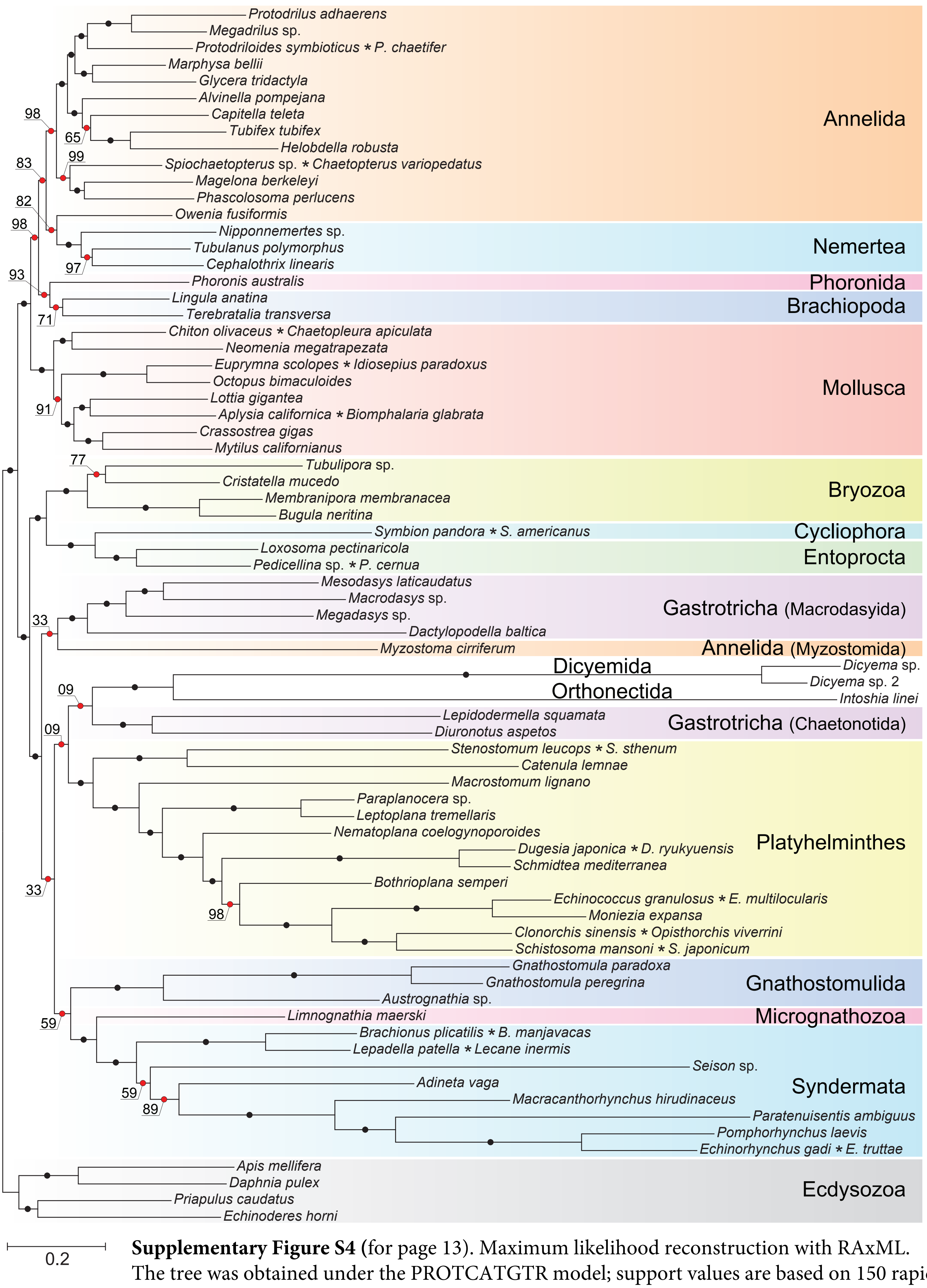

Supplementary Figure S4 (for page 13). Maximum likelihood reconstruction with RAxML. The tree was obtained under the PROTCATGTR model; support values are based on 150 rapid bootstrap replicates. Values of 100% are not shown, and corresponding nodes are marked with black dots. Chimeric operational taxonomic units include names of merged species signed with an asterisk.

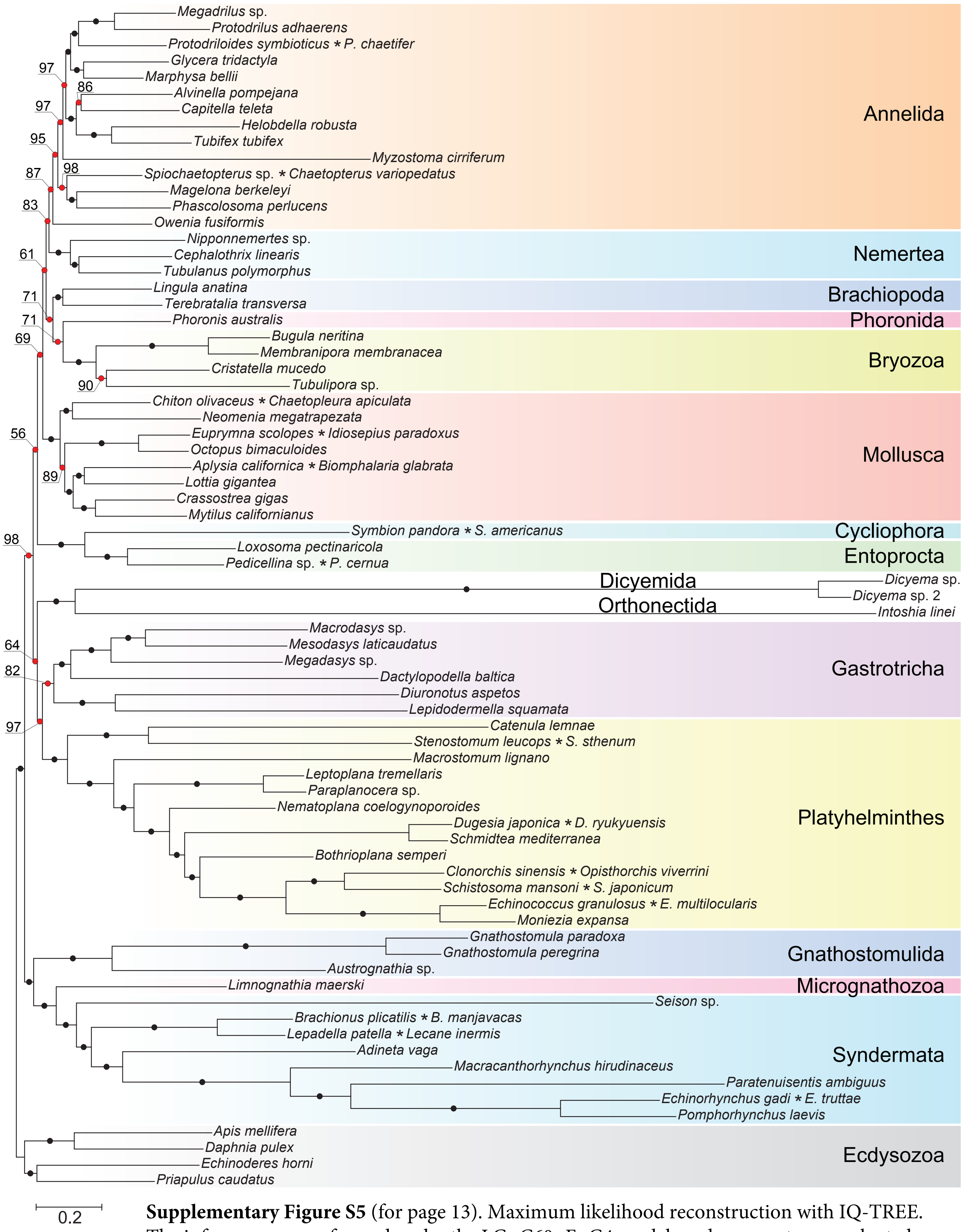

Supplementary Figure S5 (for page 13). Maximum likelihood reconstruction with IQ-TREE. The inference was performed under the LG+C60+F+G4 model; node support was evaluated with ultrafast bootstrap approximation (1000 replicates). Nodes with 100% support marked with black dots.

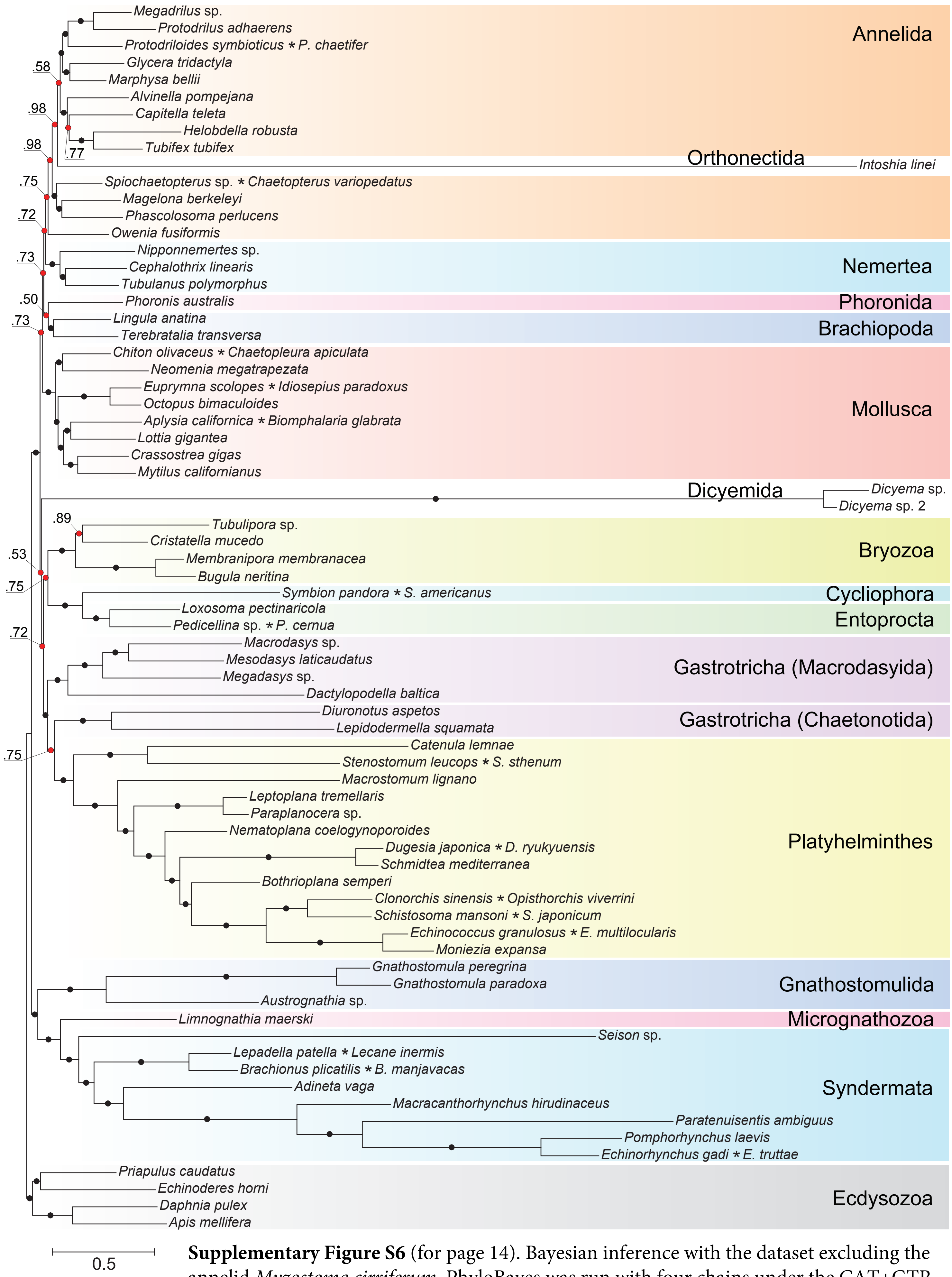

Supplementary Figure S6 (for page 14). Bayesian inference with the dataset excluding the annelid *Myzostoma cirriferum*. PhyloBayes was run with four chains under the CAT+GTR +Γ4 model. The consensus tree was constructed after 15,000 cycles with 50% burn-in (*maxdiff* =1.0). Only posterior probability values below 1.0 are shown.

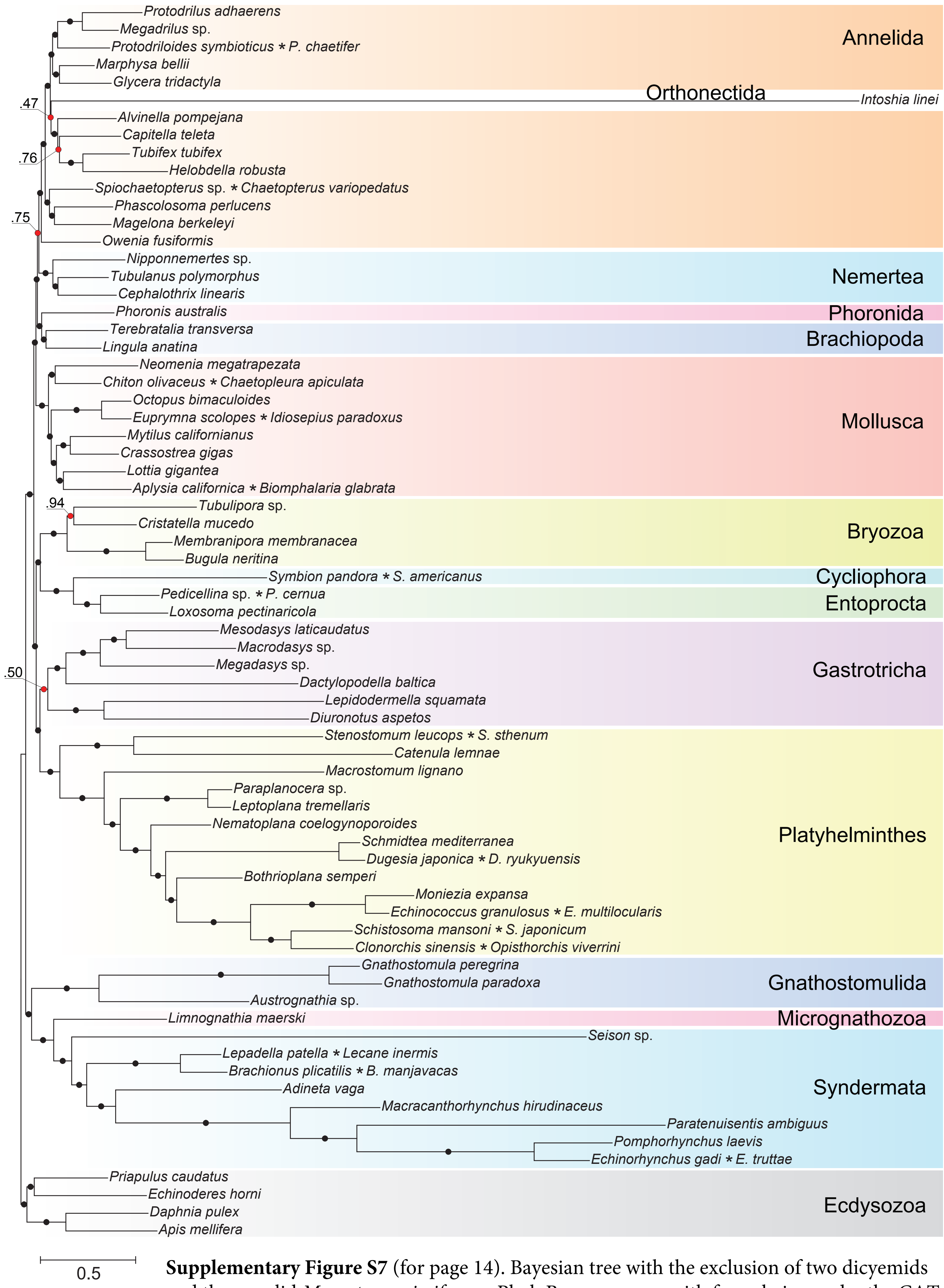

Supplementary Figure S7 (for page 14). Bayesian tree with the exclusion of two dicyemids and the annelid *Myzostoma cirriferum*. PhyloBayes was run with four chains under the CAT +GTR+Γ4 model. The consensus tree was constructed after 5,000 cycles with 50% burn-in (*maxdiff*=1.0). Only posterior probability values below 1.0 are shown.

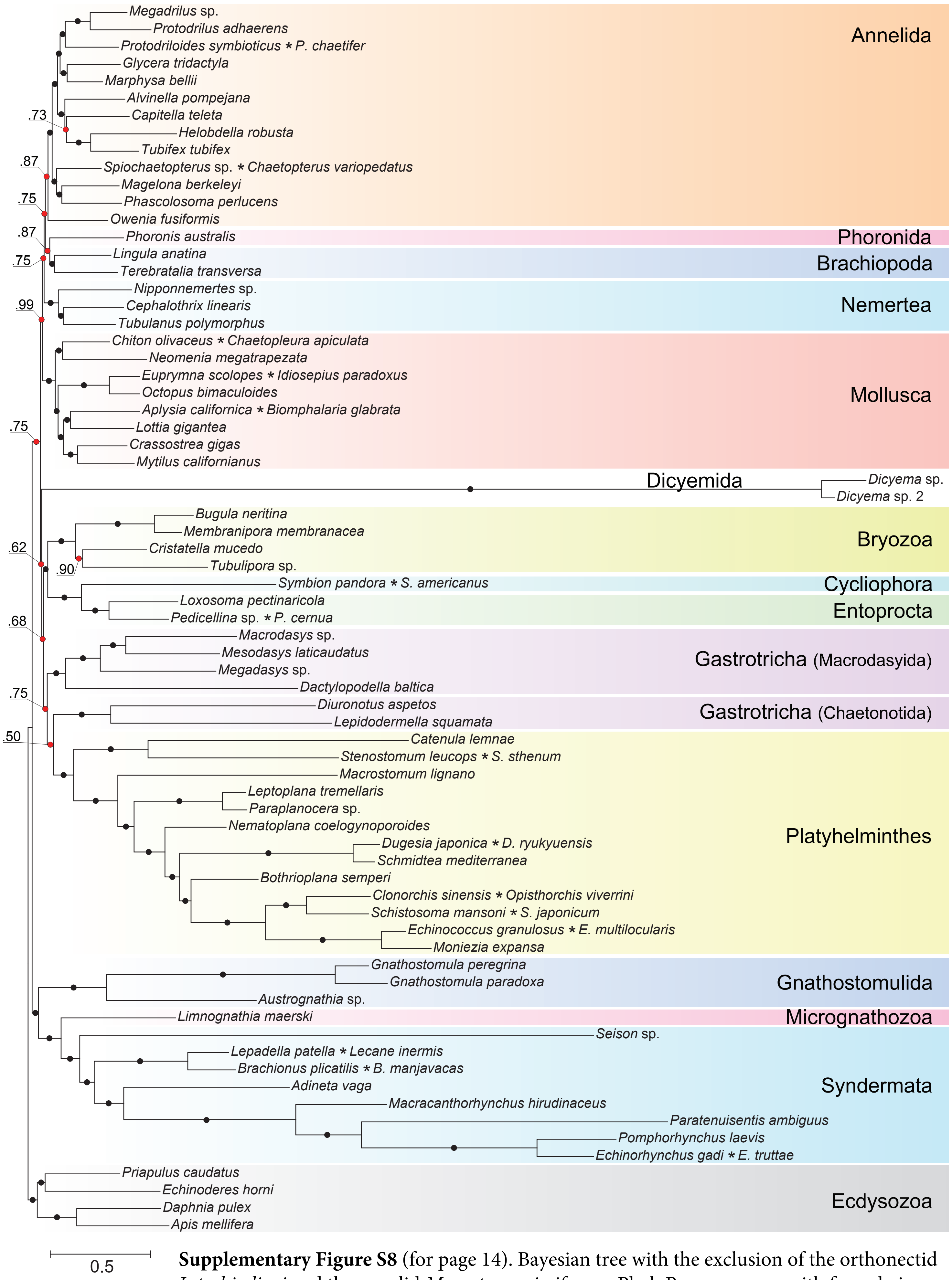

Supplementary Figure S8 (for page 14). Bayesian tree with the exclusion of the orthonectid *Intoshia linei* and the annelid *Myzostoma cirriferum*. PhyloBayes was run with four chains under the CAT+GTR+Γ4 model. The consensus tree was constructed after 5,000 cycles with 50% burn-in (*maxdiff*=1.0). Only posterior probability values below 1.0 are shown.

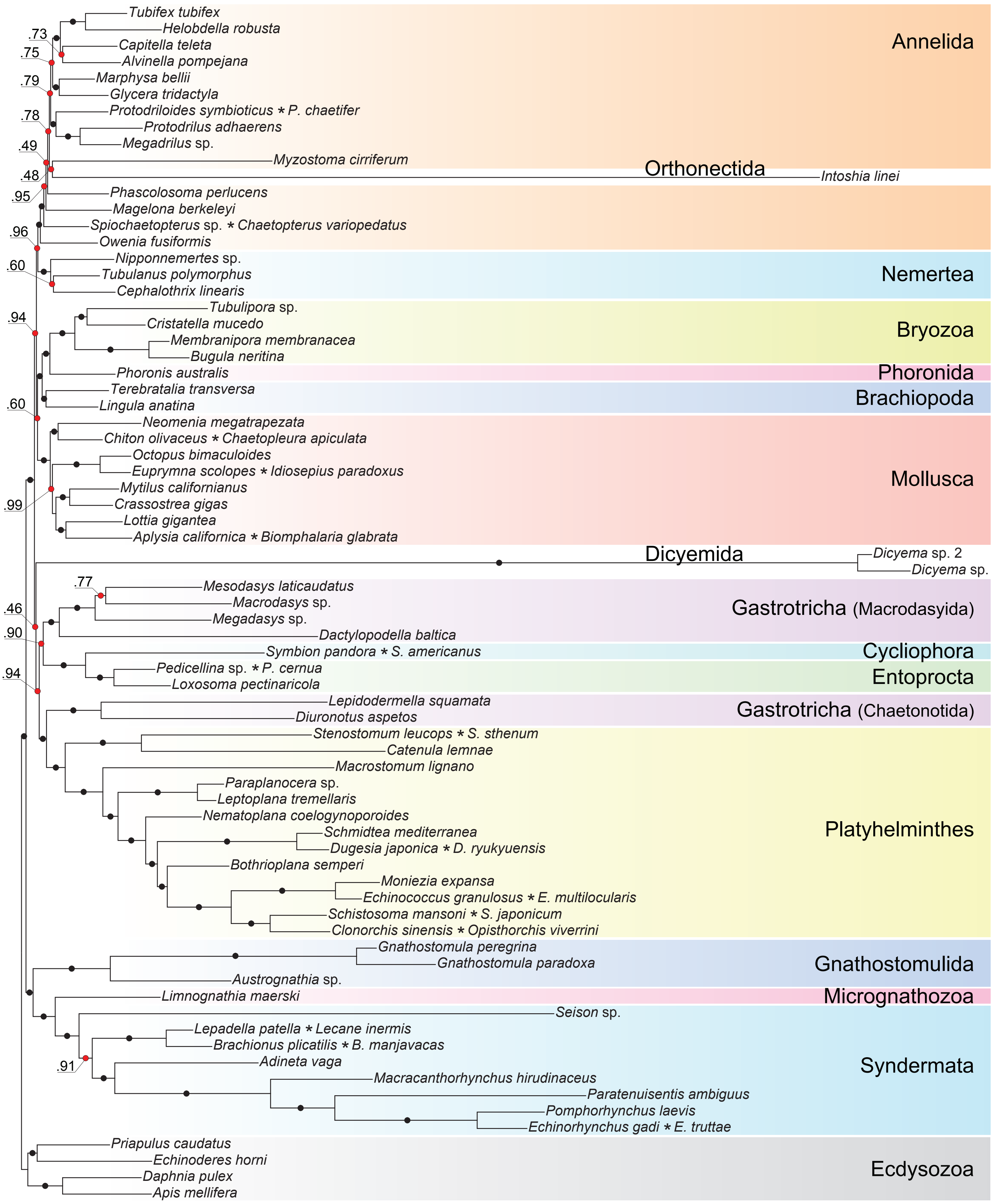

Supplementary Figure S9 (for page 14). Bayesian inference with the 35K-site dataset excluding protein alignments with high compositional heterogeneity. The inference was carried out under the CAT+GTR+Γ4 model, and the tree was generated after 50,000 cycles with 50% burn-in (*maxdiff*=0.95).

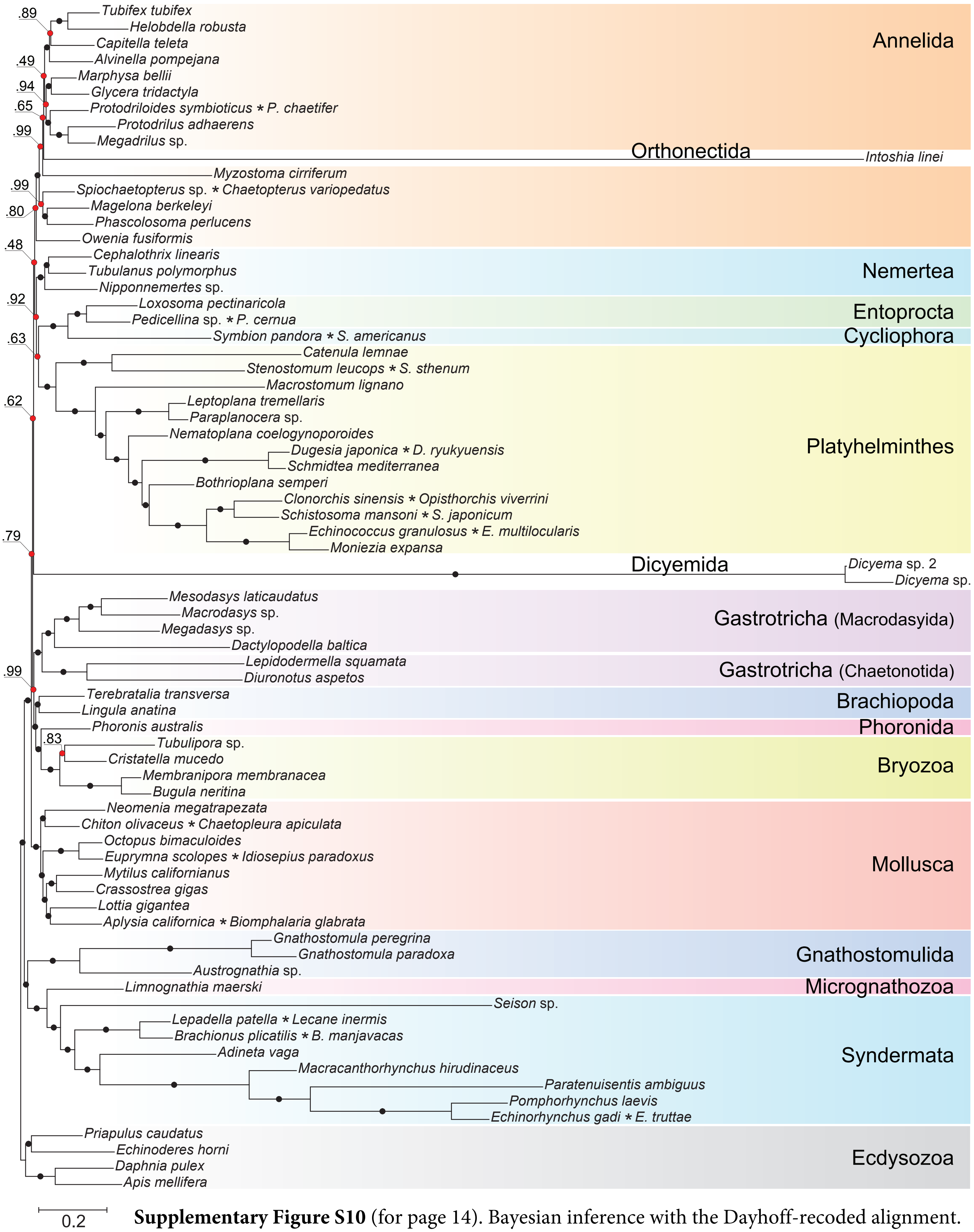

Supplementary Figure S10 (for page 14). Bayesian inference with the Dayhoff-recoded alignment. The inference was carried out under the CAT+GTR+Γ4 model; the tree was generated after 30,000 cycles with 50% burn-in (*maxdiff*=0.17).
